# Stabilization of Keratinocyte Monolayer Integrity in the Presence of Anti-Desmoglein-3 Antibodies through FcRn Blockade with Efgartigimod: Novel Treatment Paradigm for Pemphigus?

**DOI:** 10.3390/cells11060942

**Published:** 2022-03-10

**Authors:** Anna Zakrzewicz, Celina Würth, Benedikt Beckert, Simon Feldhoff, Katrien Vanderheyden, Stian Foss, Jan Terje Andersen, Hans de Haard, Peter Verheesen, Vladimir Bobkov, Ritva Tikkanen

**Affiliations:** 1 Institute of Biochemistry, Medical Faculty, University of Giessen, Friedrichstrasse 24, 35392 Giessen, Germany; anna.zakrzewicz@chiru.med.uni-giessen.de (A.Z.); celina.wuerth@dentist.med.uni-giessen.de (C.W.); benedikt.beckert@biochemie.med.uni-giessen.de (B.B.); simon.feldhoff@bio.uni-giessen.de (S.F.); 2 Argenx BV, Industriepark Zwijnaarde 7, 9052 Ghent, Belgium; kvanderheyden@argenx.com (K.V.); hdehaard@argenx.com (H.d.H.); pverheesen@argenx.com (P.V.); vbobkov@argenx.com (V.B.); 3 Department of Immunology, University of Oslo and Oslo University Hospital, Rikshospitalet, 0372 Oslo, Norway; stian.foss@medisin.uio.no (S.F.); j.t.andersen@medisin.uio.no (J.T.A.); 4 Institute of Clinical Medicine and Department of Pharmacology, University of Oslo and Oslo University Hospital, 0372 Oslo, Norway

**Keywords:** keratinocytes, epidermis, pemphigus vulgaris, autoimmune disease, autoantibodies, Fc receptor neonatal, efgartigimod

## Abstract

Pemphigus vulgaris is an autoimmune blistering disease of the epidermis, caused by autoantibodies against desmosomal proteins, mainly desmogleins 1 and 3, which induce an impairment of desmosomal adhesion and blister formation. Recent findings have shown that inhibition of immunoglobulin G binding on the neonatal Fc receptor, FcRn, results in reduced autoantibody recycling and shortens their half-life, providing a valid treatment option for PV. We have here analyzed the role of FcRn in human keratinocytes treated with antibodies isolated from pemphigus vulgaris patient or with recombinant anti-desmoglein-3 antibodies that induce pathogenic changes in desmosomes, such as loss of monolayer integrity, aberrant desmoglein-3 localization and degradation of desmoglein-3. We show that blocking IgG binding on FcRn by efgartigimod, a recombinant Fc fragment undergoing clinical studies for pemphigus, stabilizes the keratinocyte monolayer, whereas the loss of desmoglein-3 is not prevented by efgartigimod. Our data show that FcRn may play a direct role in the pathogenesis of pemphigus at the level of the autoantibody target cells, the epidermal keratinocytes. Our data suggest that in keratinocytes, FcRn may have functions different from its known function in IgG recycling. Therefore, stabilization of keratinocyte adhesion by FcRn blocking entities may provide a novel treatment paradigm for pemphigus.

## 1. Introduction

In the epidermis, resistance to mechanical stress is generated by intercellular adhesion structures such as desmosomes that are required for the integrity of the epidermis. Desmosomes contain transmembrane proteins of the cadherin family, the desmogleins (Dsg) and desmocollins (Dsc), that mediate the intercellular adhesion by interacting with their counterparts on the surface of their neighboring cells. The cytoplasmic domains of Dsg and Dsc form complexes with plaque proteins, including plakoglobin, plakophilin and desmoplakin, which mediate the interaction with the keratin filaments (for a review, see [1]).

In human diseases such as pemphigus, disruption of desmosomal adhesion results in blistering of the epidermis [2]. Pemphigus vulgaris (PV) is an autoimmune disease that manifests as flaccid blistering of the mucosa and the epidermis, caused mainly by IgG1 and IgG4 autoantibodies against the desmosomal cadherins, especially Dsg3 and Dsg1. In addition, non-desmoglein autoantibodies are frequently observed in the sera of pemphigus patients, but their contribution in pemphigus pathogenesis will require further investigation [3,4,5].

The anti-Dsg autoantibodies are usually targeted against the first cadherin homology repeat (EC1) in the extracellular domains of Dsg. Various hypotheses have been put forward to explain the mechanisms of loss of desmosomal adhesion upon autoantibody binding. While steric hindrance due to antibody binding to Dsg and Dsc plays an important role, there is ample evidence for the involvement of antibody-induced, Dsg-mediated signaling through cascades such as the mitogen-activated protein kinase (MAPK) p38 and extracellularly regulated kinase (ERK), as well as other signaling pathways (reviewed in [2,6,7,8]). For reviews on the detailed mechanisms of loss of keratinocyte adhesion and signaling in PV, please also refer to [8,9].

The autoantibodies found in PV patients (PV IgG) frequently represent a mixture of IgG antibodies towards various antigens, and over the course of the disease and due to different treatments, the antigen profile and the pathogenicity may substantially change. Therefore, although PV IgG are certainly an excellent tool for studies on PV mechanisms, their limited availability and heterogeneity over time and between patients may complicate systematic studies. Due to this, monoclonal anti-Dsg3 antibodies have been developed to facilitate studies on the mechanisms of loss of keratinocyte adhesion. These antibodies, such as the mouse monoclonal anti-Dsg3 IgG1 AK23 (mAK23), were developed by immunization of mice with recombinant Dsg3 ectodomain [10]. Further anti-Dsg antibodies were isolated by phage display from an antibody library that contained Ig variable regions of a PV patient with an active disease [11]. The single-chain antibody fragment 4B3 (also called (D3)3c/9) was isolated using a human Dsg3 ectodomain, but it also binds to human Dsg1 in an enzyme-linked immunosorbent assay (ELISA) and exhibits a binding pattern consistent with Dsg1 (all cell layers stained) in the human epidermis. Thus, although 4B3 primarily targets Dsg3, it also appears to be cross-reactive against human Dsg1 to some degree [11]. Overall, both mAK23 and 4B3 represent well-characterized, pathogenic anti-Dsg monoclonal antibodies for PV research. However, since mouse IgG exhibit only negligible binding to human neonatal Fc receptor (FcRn) [12,13], and 4B3 has so far only been used as a single-chain fragment, we here aimed to develop antibody tools that are able to interact with human FcRn and would allow the role of FcRn in the pathogenesis of PV in human keratinocytes to be studied.

The Fc receptor family members are important for the regulation of various cellular functions of antibody molecules, as demonstrated by the findings showing that genetic polymorphisms in Fc receptors or in the IgG molecules that affect their mutual affinity may modulate the tissue distribution of autoantibodies in pemphigus [14,15]. In particular, FcRn has recently been established as a therapeutic target in pemphigus [16,17]. FcRn shows high structural similarity to the MHC (major histocompatibility complex) class I molecule, comprising a single-pass transmembrane chain and β_2_ microglobulin that is also found in MHC I. FcRn is involved in the recycling of IgG-type antibodies and albumin, prolonging their half-life [18,19,20]. Binding of albumin and IgG to FcRn is mediated by separate binding sites and with different stoichiometry, as albumin binding is equimolar, whereas IgG molecules are bound by two FcRn dimers [21,22,23,24]. FcRn binds IgG in the acidic pH of endosomal compartments, whereas the decreased FcRn affinity in the physiological pH results in IgG release after the complex has reached the cell surface [25,26]. Thus, degradation of IgG in lysosomes is reduced by FcRn-mediated recycling of IgG molecules [27,28,29].

In addition to its role in IgG and albumin recycling in the blood, FcRn has also been shown to be involved in bidirectional transcytosis of IgG across various epithelia [30,31,32,33,34]. Furthermore, FcRn appears to mediate the lysosomal degradation of autoantibody-antigen immune complexes (IC) in the target cells of the autoantibodies [35,36,37]. A recent review by Qi and Cao is recommended for a thorough summary of FcRn functions [38]. Although it has been shown that keratinocytes endogenously express FcRn [39], a potential direct role of FcRn in the pathogenesis of pemphigus and in the transport of autoantibodies in keratinocytes requires further studies.

Efgartigimod is a human IgG1 Fc fragment that has been engineered for increased affinity to FcRn, while retaining some of the pH dependent FcRn binding characteristics [31,40]. Fc region-mediated binding of efgartigimod to FcRn blocks the binding of IgG, resulting in targeted reduction of all IgG subtypes without impacting the levels of other immunoglobulin isotypes, or IgG production [40,41]. In a phase 1 study with healthy volunteers, phase 2 and 3 studies in patients with myasthenia gravis, and a phase 2 study in primary immune thrombocytopenia (ITP), efgartigimod was well tolerated and led to a reduction of IgG antibodies, which was associated with statistically significant clinical improvements [40,41,42,43]. PV is mediated by autoantibodies of the IgG type (mainly IgG4 and IgG1), and efgartigimod has recently been shown to be a promising therapeutic approach for PV, as efgartigimod produced an early clinical response in a phase 2 study in PV patients [16]. In addition, the full-length FcRn blocking IgG4, ALXN1830, has recently been suggested to be suitable for the treatment of pemphigus [17].

The research on the mechanisms of PV has focused on the characterization of the loss of desmosomal adhesion in keratinocytes due to autoantibodies against Dsg, whereas the potential function of FcRn in mediating the pathogenic effects of anti-Dsg autoantibodies in keratinocytes has not been addressed so far. The main purpose of the present study was to investigate the role of autoantibody-FcRn interaction in the pathogenic effects of anti-Dsg autoantibodies in pemphigus and to explore the mechanisms of efgartigimod-mediated blockade of FcRn in human keratinocytes. The rapid clinical response observed in PV patients treated with efgartigimod [16] triggered us to investigate the function of FcRn and efgartigimod in human keratinocytes. In this study, we have produced and characterized novel, recombinant anti-Dsg3 IgG antibodies that are based on the well-characterized mAK23 and 4B3 antibodies, but carry a human Fc region capable of binding to FcRn in human keratinocytes. These reformatted antibodies exhibited a high pathogenicity in human keratinocytes, but blocking of FcRn by efgartigimod efficiently prevented the fragmentation of keratinocyte monolayers induced by the engineered pathogenic antibodies. Furthermore, 4B3 treatment resulted in a loss of FcRn that was rescued by efgartigimod. Our data thus provide evidence that FcRn acts directly at the level of epidermal keratinocytes in the context of PV, and that its function is not based merely on autoantibody recycling. Since efgartigimod treatment efficiently rescues the loss of keratinocyte adhesion upon anti-Dsg3 antibody and PV IgG treatment, our data strongly support the use of efgartigimod in the treatment of PV and suggest that stabilization of keratinocyte adhesion may present a novel treatment paradigm for pemphigus.

## 2. Materials and Methods

### 2.1. Cell Culture

The human keratinocyte cell line hTert/KER-CT (ATCC^®^, CRL4048) was purchased from LGC Standards GmbH (Wesel, Germany) and cultured as described [44]. The cells were maintained at 37 °C and 5% CO_2_ in basal Keratinocyte Growth Medium 2 (KGM2, PromoCell, Heidelberg, Germany) supplemented with 4 µL/mL bovine pituitary extract, 0.125 ng/mL epidermal growth factor (recombinant human), 5 µg/mL insulin (recombinant human), 0.33 µg/mL hydrocortisone, 0.39 µg/mL epinephrine, 10 µg/mL transferrin (recombinant human), 0.05 mM CaCl_2_ (all from PromoCell) and 30 µg/mL gentamicin sulphate (Serva, Heidelberg, Germany). For experiments under high calcium conditions, the cells were grown in KGM2 with 2 mM CaCl_2_. The human epithelial cell line T84 was maintained at 37 °C in a humidified incubator under 5% CO_2_ in Dulbecco’s modified Eagle’s medium (DMEM)/F-12 medium (1:1), supplemented with 20% heat-inactivated fetal bovine serum, 50 U/mL streptomycin and 50 U/mL penicillin.

### 2.2. Antibody Production and Purification

The hybridoma cell line producing the monoclonal mouse AK23 (mAK23) anti-Dsg3 antibody (Ig1, kappa chain) against mouse and human Dsg3 was a generous gift from M. Amagai and has been previously described [10]. Hybridoma cells were cultured as suspension in RPMI 1640 medium supplemented with 10% fetal bovine serum, 1% penicillin/streptomycin, 1% non-essential amino acids, 1% sodium pyruvate (all from Gibco/Life Technologies, Carlsbad, CA, USA) and 55 µM β-mercaptoethanol (Sigma-Aldrich, Munich, Germany) at 37 °C in a humidified atmosphere with 5% CO_2_. For mAK23 production, the cells were seeded into a medium containing 40% of the culture medium and 60% of ISF-I hybridoma medium (Biochrom, Berlin, Germany) and grown for ten days. The antibody fraction was purified from the culture medium using Protein G Sepharose 4 Fast Flow (Sigma Aldrich, Taufkirchen, Germany) and concentrated in PBS, as described in [44].

The anti-Dsg3 antibody mAK23 was reformatted by cloning the mouse DNA sequences of the variable fragments of the heavy and light chain [45] into human IgG4 and human kappa light chain backbones, respectively. The resulting chimeric hAK23 antibody carrying human CL, CH1, and hinge regions, as well as a human Fc part that allows binding to human FcRn, was produced in HEK293 cells. The recombinant antibody was purified by protein A affinity chromatography, followed by size exclusion chromatography (Absolute Antibody Ltd., Redcar, UK).

The sequences of the variable regions of the 4B3 antibody were kindly provided by Dr. John R. Stanley. Recombinant 4B3 was produced in a fully human IgG1 backbone in Chinese hamster ovarian (CHO-K1) cells and purified by protein A affinity chromatography (evitria AG, Zurich, Switzerland). Efgartigimod, Fc-WT, Fc-IHH, and human isotype control IgG1 and IgG4 antibodies were also produced by evitria AG (Switzerland), as described previously [40].

The sequences for the heavy and light chains of the anti-FcRn antibody were retrieved from the patent application WO2012/167039, and the antibody was produced as described previously [46].

### 2.3. Dispase-Based Monolayer Dissociation Assay

Dispase-based monolayer dissociation assays were performed as previously described [44]. The cells were seeded in 24-well plates and cultured in KGM2 medium containing 0.05 mM CaCl_2_ until confluent. After reaching confluence, the culture medium was exchanged to KGM2 with 2 mM CaCl_2_ for a further 24 h. The cell monolayers were incubated with either mAK23 (75 µg/mL), hAK23 (0.1–50 µg/mL) or 4B3 (25–100 µg/mL) for 24 h at 37 °C and 5% CO_2_. In all experiments, a treatment with appropriate human control-IgG was included.

The cells were washed with Hanks’ Balanced Salt Solution (HBSS, Cat. 14025-050, Gibco, Karlsruhe, Germany) and incubated with 2.5 U/mL Dispase II (Cat. 04942078001, Roche, Mannheim, Germany) solution for about 25 min until the monolayers were completely detached. Released monolayers were washed with HBSS and incubated with 3-(4,5-dimethylthiazol-2-yl)-2,5-diphenyltetrazolium bromide (MTT, at final concentration 0.25 mg/mL, Sigma-Aldrich, Taufkirchen, Germany) for 15 min. For fragmentation, mechanical stress was applied to the monolayer by pipetting up and down using a 1 mL plastic pipette tip. The same conditions were used for all samples. All samples were prepared in triplicates. The fragments were fixed using 4% paraformaldehyde and counted automatically using the ImageJ software [47,48].

### 2.4. Cell Lysis, Gel Electrophoresis and Western Blot

For Western blot analyses, the cells were lysed using lysis buffer (50 mM Tris pH 7.4; 150 mM NaCl; 2 mM EDTA; 1% NP-40) supplemented with a protease inhibitor cocktail (Sigma Aldrich, Taufkirchen, Germany). Protein concentration was determined using Bradford assay (Bio-Rad, Munich, Germany). Equal amounts of proteins were separated by SDS-PAGE and transferred to a nitrocellulose membrane. Precision Plus Protein™ Dual Color standards (Bio-Rad, Munich, Germany) were used to determine the molecular weight. Membranes were blocked for 1 h with 5% low fat milk (Roth, Karlsruhe, Germany), followed by overnight incubation at 4 °C with primary antibodies: anti-Dsg1 (B-11) (1:1000, Cat. sc-137164, Santa Cruz Biotechnology, Heidelberg, Germany), anti-Dsg3 (5H10) (1:1000, Cat. sc-23912, Santa Cruz Biotechnology), anti-FcRn (1:1000, Cat. NBP1-89128, Novus Biologicals/Bio-Techne, Wiesbaden, Germany) or anti-GAPDH (1:10,000, Cat. ab-8245, Abcam, Cambridge, MA, USA). After extensive washing, the membranes were further incubated for 1 h at room temperature with appropriate horseradish peroxidase (HRP)-conjugated secondary antibodies (Dako, Glostrup, Denmark). The signals were detected by enhanced chemiluminescence assay on a roentgen film. To quantify the signals, densitometric analysis of scanned films was performed using the ImageJ software [47]. Intensity of the signal was normalized to GAPDH.

### 2.5. Sequential Detergent Extraction

Sequential detergent extraction was carried out according to Stahley et al., with some modifications [49]. Briefly, hTert cells were seeded in 6-well plates and cultured in KGM2 medium containing 0.05 mM CaCl_2_ for at least four days until confluent. After that, the medium was exchanged to KGM2 with 2 mM CaCl_2_, and the cells were cultured for a further 24 h. Thereafter, the cells were treated with efgartigimod (25 µg/mL) for 30 min, or left untreated. For treatment with antibodies, 4B3 (at concentration 50 µg/mL) or a matching IgG1 control antibodies were applied for the following 24 h. Finally, the cells were lysed using Triton buffer (containing 1% Triton X-100, 10 mM Tris-HCl pH 7.5, 140 mM NaCl, 5 mM EDTA, 1 mM PMSF, 1 µg/mL Leupeptin, 1 µg/mL Pepstatin A). Cell lysates were centrifuged at 14.000× *g* for 30 min. The supernatant containing the Triton-soluble proteins was collected. The Triton-insoluble proteins that remained in the pellet were subsequently extracted using SDS/urea buffer (containing 1% SDS, 8 M urea, 10 mM Tris HCl pH 7.5, 140 mM NaCl, 5 mM EDTA, 2 mM EGTA). The Triton-soluble and insoluble fractions were processed for Western blot analysis. To control the purity of the extracted fractions and to enable quantification of Dsg1 and Dsg3, GAPDH level was used as a reference. The intensity of the signal in Triton-soluble fraction was normalized to GAPDH.

### 2.6. Immunofluorescence

For fluorescent staining, 3 × 10^4^ hTert cells were seeded on glass coverslips and cultured for at least three days in KGM2 with 0.05 mM CaCl_2_. For differentiation, the medium was exchanged to KGM2 with 2 mM CaCl_2_ for 24 h. The cells were fixed in methanol for 8 min at –20 °C. Subsequently, the cells were treated with 1% BSA for 30 min, and staining with the primary antibodies was carried out for 1 h at room temperature in 1% BSA/PBS. The following antibodies were used: anti-Dsg3 (5H10, Santa Cruz Biotechnology), hAK23 or 4B3 (both at 2 µg/mL), or mAK23 (8 µg/mL). Alternatively, after 24 h culture in KGM2 with 2 mM CaCl_2_, the cells were incubated with mAK23 (10 µg/mL), hAK23 (12.5 µg/mL) or 4B3 (50 µg/mL) for further 24 h, followed by fixation with methanol. For the detection of bound primary antibodies, Alexa Fluor 488-coupled donkey anti-mouse IgG (1:300, Cat. A-21202, Invitrogen, Carlsbad, CA, USA) or anti-human IgG (1:300, Cat. 709-545-149, Jackson ImmunoResearch, Ely, UK) secondary antibodies were applied for 1 h in 1% BSA/PBS.

For visualization of the pathogenic effect, the cells were incubated for 24 h at 37 °C with Alexa Fluor 488-coupled hAK23 (12.5 µg/mL) or 4B3 (50 µg/mL) or an isotype matched human IgG4 (12.5 µg/mL, Cat. DDXCHO1A488-100, Novus Biologicals/Bio-Techne, Wiesbaden, Germany) or IgG1 (50 µg/mL, Cat. DDXCHO4A488-100, Novus Biologicals) antibodies. After methanol fixation, the control IgG-treated cells were blocked with 1% BSA for 30 min and stained with the monoclonal anti-Dsg3 5H10 antibody (1:100) for 1 h at room temperature, followed by an incubation with donkey anti-mouse IgG Alexa Fluor 546-coupled secondary antibodies (1:100, Cat. A10036, Invitrogen, Carlsbad, CA, USA). For the double staining of hAK23 and mAK23, the cells were first stained with Alexa Fluor 488-coupled hAK23 (16 µg/mL), and then with mAK23 (8 µg/mL) that was detected with an Alexa Fluor 546-coupled secondary anti-mouse antibody. All samples were mounted using Roti-Mount FluorCare DAPI mounting medium (Carl Roth, Karlsruhe, Germany). The samples were analyzed using a Zeiss LSM710 Confocal Laser Scanning Microscope (Carl Zeiss, Oberkochen, Germany) or an Aurox Clarity laser-free spinning disc confocal microscope (Aurox Ltd., Oxfordshire, UK).

### 2.7. Anti-Dsg3 ELISA

Conditioned media of hTert cells treated with 12.5 µg/mL hAK23 or 50 µg/mL 4B3 and efgartigimod were collected at time points 0 h and 24 h and stored at −20 °C. The anti-Dsg3 antibody titers in the medium were determined with the MESACUP-2 test Dsg3-based enzyme-linked immunosorbent assay (ELISA; MBL, Nagoya, Japan), according to the manufacturer’s instructions and as described previously [50,51,52]. Briefly, the samples were diluted 1:200 (hAK23) or 1:800 (4B3) in assay diluent, and the antibody concentrations were determined against a calibration curve with the pure hAK23 (7.8–125 ng/mL) or 4B3 (1.6–200 ng/mL).

### 2.8. AK23 Competition ELISA and Apparent Affinity Determination

The MESACUP-2 test Dsg3 ELISA (MBL, Nagoya, Japan) was used according to the manufacturer’s instructions, with adaptations. For the hAK23 competition ELISA, hAK23-IgG4 (tracer), fixed at 0.13 nM final concentration, was combined with a titration series of the competing antibody, mAK23-IgG1 (40 nM starting concentration, 1:3 dilution steps, 9 points, assay diluent blank). For the mAK23 competition ELISA, a fixed mAK23-IgG1 (tracer) final concentration (0.13 nM) was combined with a titration series of the competing antibody, hAK23-IgG4 (equivalent to the hAK23 competition ELISA). Antibody dilutions were prepared in assay diluent. hAK23-IgG4 was detected with an HRP-conjugated goat anti-human IgG antibody of the MESACUP-2 kit (MBL, Nagoya, Japan), whereas mAK23-IgG1 was detected with an HPR-conjugated, Fc-specific rabbit anti-mouse IgG-HRP (Jackson 315-035-008, 80 ng/mL final concentration), diluted in assay diluent. Both assay diluent and the competing antibody were included as controls at 0.83 nM final concentration to evaluate the nonspecific binding and cross-reactivity of the respective detection antibody. The substrate was incubated for 45 min.

Apparent affinity was determined as follows: The MESACUP-2 test Dsg3 ELISA (MBL, Nagoya, Japan) was used according to the manufacturer’s instructions with adaptations. Briefly, a dilution series of anti-Dsg3 autoantibodies was prepared in assay diluent, hAK23-IgG4 (0.013 nM to 0.833 nM), mAK23-IgG1 (0.013 nM to 0.417 nM), and 4B3-IgG1 (0.010 nM to 1.333 nM). hAK23 and 4B3 were detected with an HRP-conjugated goat anti-human IgG antibody and mAK23 with an HRP-conjugated, Fc-specific rabbit anti-mouse IgG, diluted in assay diluent (Jackson 315-035-008; 106.7 ng/mL final concentration). The substrate was incubated for 30 min.

### 2.9. RNA Isolation and cDNA Synthesis

Total RNA was extracted from hTert cells using Trizol reagent (Invitrogen, Karlsruhe, Germany) as described previously [44]. Then, 1.5 µg of total RNA was reverse transcribed using M-MuLV reverse transcriptase (New England Biolabs, Frankfurt, Germany) and 150 fmol oligo(dT) primers. The reaction was carried out at 37 °C for 2 h in a total volume of 45 µL.

### 2.10. Quantitative Real-Time PCR

The mRNA levels of *DSG3* and *FCGRT* (the gene coding for FcRn heavy chain) were assessed by quantitative real-time PCR. Reactions were carried out in the CFX Connect Real-Time OCR Detection System (Bio-Rad, Munich, Germany) using iTaq^TM^ Universal SYBR Green Supermix (Bio-Rad, Munich, Germany). Quantitative changes were calculated using the ∆CT method. The geometric mean of the reference genes *RPL13a*, *GAPDH*, *YWHAZ* and *B2M* was used for normalization. The primer sequences for the reference genes and *DSG3* were as published before [44]. For the amplification of the FcRn encoding cDNA, the following primers were used: (forward) 5′-TGGAGAAGGATGAGGAGTGG-3′ and (reverse) 5′-GGTGGCTGGAATCACATTTA-3′. The annealing temperature for all primers was 60 °C. Each sample was assessed in duplicates.

### 2.11. Transcytosis Assay

Transwell filters (1.12 cm^2^) with collagen-coated polytetrafluoroethylene (PTFE) membrane and 0.4 m pore size (Corning/Costar; Fisher Scientific, Oslo, Norway) were incubated overnight in the growth medium before 1.0 × 10^6^ T84 cells were seeded in each well. Transepithelial electrical resistance (TEER) was measured daily using a MILLICELL-ERS volt-ohm meter. The cells were cultured for 5 to 7 days until they reached a TEER value of 1500–1900 Ω × cm^2^. Growth medium was exchanged daily. Prior to the experiments, the T84 monolayers were washed and incubated for 1 h in HBSS. Then, 200 nM of the Fc fragment variants or corresponding full-length antibodies or an anti-FcRn antibody [46] (all diluted in 200 μL HBSS) were added to the apical side, followed by an incubation for 4 h and collection of 500 μL HBSS from the basolateral reservoir.

### 2.12. Quantification of Transcytosed Fc Fragment and IgG Variants by ELISA

For transcytosis assay, 96-well enzyme immunoassay plates (Corning/Costar; Fisher Scientific, Oslo, Norway) were coated with polyclonal anti-human IgG (whole molecule) antibody diluted to 1 µg/mL in phosphate buffered saline (PBS). The plates were blocked with 4% low-fat milk in PBS for two hours at room temperature, followed by washing 4 times with PBS/0.05% Tween 20. Basolateral media samples from the transcytosis experiments were added to the wells and incubated for 1 h at room temperature in duplicates, before washing as above. Bound antibodies were detected using an alkaline phosphatase conjugated, goat polyclonal anti-human IgG (Fc-specific) antibody. Binding was visualized by addition of 100 µL phosphatase substrate, and the 405 nm absorbance values were recorded using a Tecan Sunrise spectrophotometer. The amounts of transported Fc fragments and IgG were calculated by interpolating the average absorbance values obtained from each individual well towards standard curves of each individual Fc fragment (20.0–0.009 nM) or IgG (6.670–0.003 nM) using the sigmoidal dose-response (variable slope) non-linear regression model of GraphPad Prism.

### 2.13. PV Patient and IgG Isolation

A baseline serum sample from a PV patient with newly diagnosed mucosal-dominant PV from the open-label, phase 2 study of efgartigimod in pemphigus was used for PV IgG isolation [16]. The anti-Dsg-3 autoantibody level of this sample was 500 RU/mL (EUROIMMUN, Lübeck, Germany). The IgG fraction of the serum was isolated as described in [44,53]. The IgG fraction was dialyzed against PBS and used at 150 µg/mL. Normal human (NH) IgG was isolated from healthy controls and used at the same concentration.

### 2.14. Statistical Analysis

All experiments were performed at least three times, or as indicated in the figure legends. The data are expressed as mean ± SD. Statistical analyses were performed with GraphPad Prism 5.0 (GraphPad Software, Inc., San Diego, CA, USA). For multiple comparisons with a control group, One-way analysis of variance (ANOVA) with Dunnett’s post-test was used. In some cases, two-way ANOVA with Bonferroni’s post-test was performed, as specified in the figure legends. 

## 3. Results

### 3.1. Reformatted Antibodies hAK23 and 4B3 Recognize Dsg3 in Indirect Immunofluorescnce of Human Keratinocytes

Since mouse IgG exhibit only negligible binding to human FcRn [12], we aimed at developing tools based on human or chimeric IgGs that would allow investigation of a potential role of FcRn in the pathogenicity of anti-Dsg3 antibodies. For this, the well-characterized anti-Dsg3 mouse antibody, mAK23 [10], was reformatted as a chimeric IgG4 antibody containing human Fc region (hAK23, see Section 2.2 for details). In addition, another previously described pathogenic anti-Dsg3 antibody, 4B3, originally isolated from a PV patient [11,54], was produced as a recombinant human IgG1. The monoclonal antibodies hAK23 and mAK23 showed similar binding affinities and were able to compete for the binding to recombinant Dsg3 in an ELISA assay (Supplementary Figure S1).

**Figure 1:**
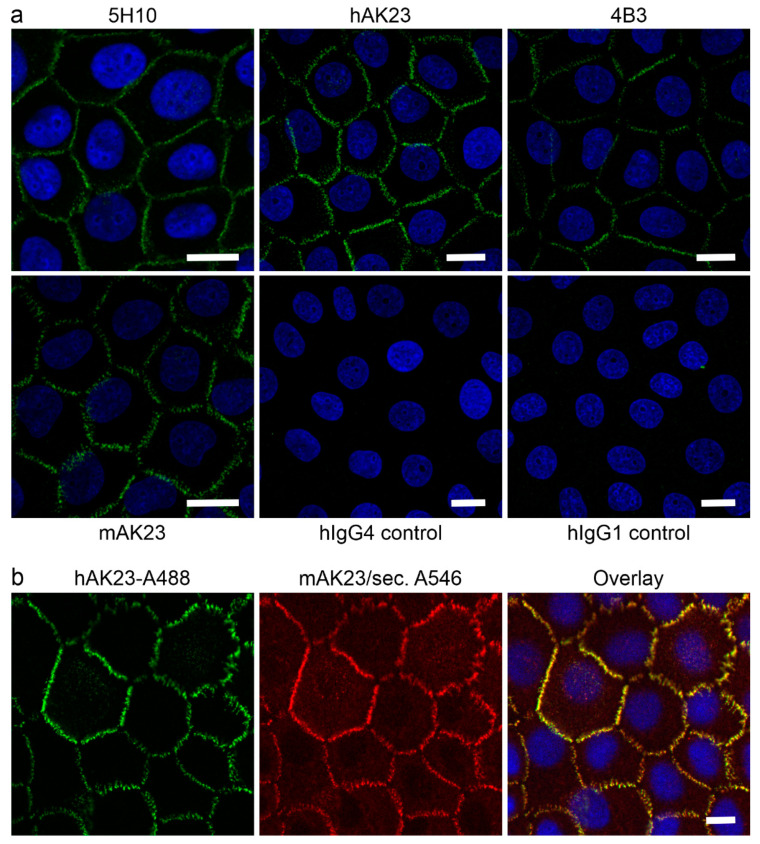
Staining pattern of Dsg3 in human hTert keratinocytes with the hAK23 and 4B3 antibodies is highly similar to mAK23 and 5H10 antibodies. Human hTert keratinocytes were cultured on coverslips in KGM2 medium with 0.05 mM CaCl_2_ for at least three days, and then switched to KGM with 2 mM CaCl_2_ for 24 h. After fixation with methanol, the cells were stained with recombinant anti-Dsg3 antibodies hAK23 or 4B3, as indicated. Staining with the anti-Dsg3 5H10 antibody, mAK23 or isotype-matched human control IgGs (hIgG) were included as controls. (**a**) Post-fixation staining with the indicated antibodies. Fluorochrome-coupled secondary antibodies (anti-mouse or anti-human Alexa Fluor 488, green) were used for the detection of the bound primary antibodies. (**b**) Double staining with Alexa Fluor 488-coupled hAK23 (16 µg/mL, green) and mAK23 (8 µg/mL) detected with a secondary antibody coupled with Alexa Fluor 546 (red). The coverslips were mounted using a mounting medium with DAPI (blue). Representative images from one out of three independent experiments are shown. Scale bar: 20 µm.

To show that the recombinant antibodies hAK23 and 4B3 exhibit a Dsg3-like staining pattern, a post-fixation immunofluorescence staining of human hTert keratinocytes was performed. Mouse monoclonal anti-Dsg3 antibodies 5H10 and mAK23, as well as isotype-matched human IgGs (hIgG4 for hAK23 and hIgG1 for 4B3) were used as controls. All anti-Dsg3 antibodies showed a typical Dsg3 immunostaining pattern at the cell–cell borders, whereas the control hIgGs showed only a very low background signal (Figure 1a). Thus, the reformatted anti-Dsg3 antibodies hAK23 and 4B3 exhibited a highly similar staining pattern as mAK23 and 5H10 antibodies. Double staining with fluorochrome-coupled hAK23 and mAK23 that was detected with a fluorochrome-coupled secondary antibody revealed nearly identical staining patterns and almost perfect overlap, as expected for two antibodies that have identical antigen binding regions (Figure 1b). Control staining with Alexa Fuor 488-coupled hAK23 and the secondary anti-mouse Alexa Fluor 546-coupled antibody showed that the secondary anti-mouse antibody does not cross-react with hAK23 (Supplementary Figure S2).

### 3.2. Recombinant Anti-Dsg3 Antibodies hAK23 and 4B3 Induce Acantholysis, Reduce Monolayer Integrity and Alter Dsg3 Localization in Human Keratinocytes

**Figure 2:**
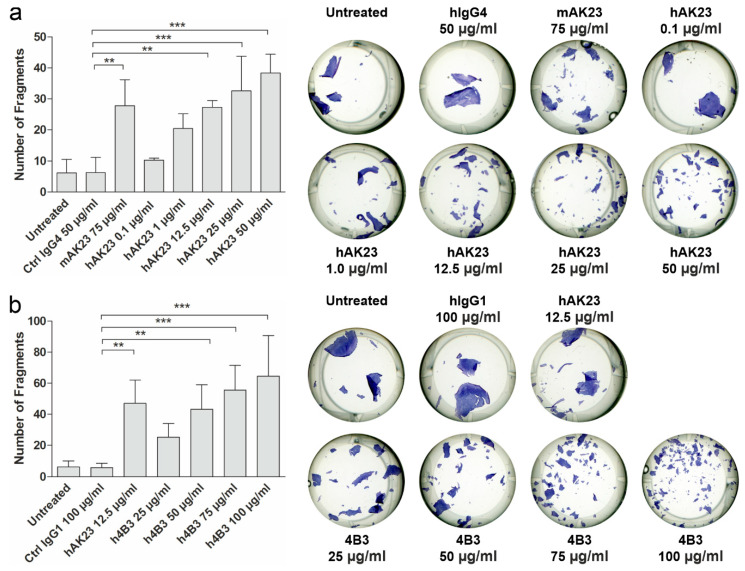
The anti-Dsg3 antibodies hAK23 and 4B3 are pathogenic in a monolayer dissociation assay. hTert keratinocytes were plated on 24-well plates, grown in KGM2 medium with 0.05 mM CaCl_2_ until confluent and then switched to KGM2 with 2 mM CaCl_2_ for 24 h. Thereafter, the cells were treated for 24 h with different amounts of the indicated antibodies (**a**) hAK23 or (**b**) 4B3, to induce acantholysis. As controls, mock incubation (untreated), an isotype-matched hIgG (hIgG4, 50 µg/mL), or hIgG1 (100 µg/mL) and mAK23 (75 µg/mL) were used. A monolayer fragmentation assay was performed, and the number of fragments was quantified using the ImageJ software. Representative images for each treatment are shown. The error bar represents the SD of the mean values obtained from at least four independent experiments, each of which was performed in triplicate. Statistical analysis was done using one-way analysis of variance (ANOVA) with Dunnett’s post-test. Statistically significant differences are indicated by asterisks. ** = *p* ≤ 0.01; *** = *p* ≤ 0.001.

The pathogenic effect of the recombinant anti-Dsg3 antibodies was tested in a monolayer dissociation assay in hTert keratinocytes (Figure 2). The cells were grown as a dense monolayer as described above and in [44]. The monolayers were incubated with the indicated antibodies for 24 h to induce acantholysis. As controls, mock incubation (untreated), isotype-matched hIgG and mAK23 were used.

Different amounts of the recombinant antibodies were used to study dose-dependence of the monolayer dissociation. The monolayers were detached by dispase treatment. After staining with MTT, the monolayers were dissociated by mechanical stress (repeated shearing with a pipette). The data show that the recombinant antibodies hAK23 and 4B3 induced a dose-dependent fragmentation of the monolayer (Figure 2a,b). Although mAK23 and hAK23 are targeted against the same epitope in Dsg3, different amounts were required to induce the same number of fragments. This may be due to the different manufacturing processes. These antibodies are also of different subtypes, with hAK23 belonging to the IgG4 subclass, whereas mAK23 is mIgG1. For further experiments, a working concentration was chosen that gave a similar response as 75 µg/mL mAK23: 12.5 µg/mL for hAK23, and 50 µg/mL for 4B3. The control hIgGs of the matching subclasses were used at the respective concentrations.

Treatment of keratinocytes with pathogenic anti-Dsg antibodies results in reorganization of Dsg at the plasma membrane [55,56]. Treatment of hTert cells with the pathogenic mAK23, hAK23, or 4B3 or with the control antibodies for 6 or 24 h resulted in characteristic changes in Dsg3 localization (Supplementary Figure S2). After 6 h, hAK23, mAK23 and 4B3 showed a disruption of the Dsg3 staining at the cell borders, which was further abrogated upon 24 h, whereas the control hIgGs exhibited no staining. Upon treatment with the pathogenic anti-Dsg3 antibodies, Dsg3 exhibited a more diffuse and broad staining as compared to the post-fixation staining, with some intracellular structures (compare Figure 1 with Supplementary Figure S2), indicating that desmosomal morphology was altered due to a change in Dsg3 arrangement at the plasma membrane.

### 3.3. Treatment with hAK23 and 4B3 Results in Reduced Dsg3 Amount, and 4B3 Induces FcRn Depletion

**Figure 3:**
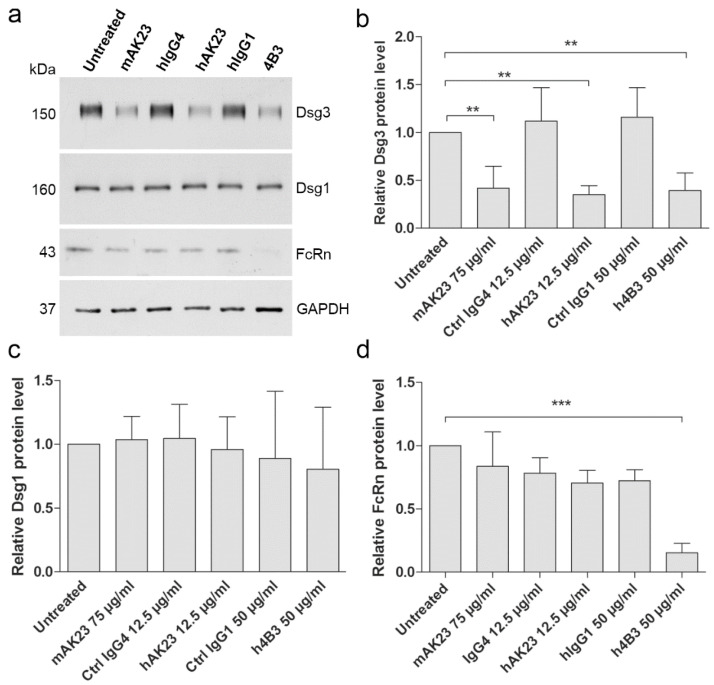
Treatment of keratinocytes with hAK23 and 4B3 antibodies results in Dsg3 depletion, and 4B3 abrogates FcRn protein level. (**a**) hTert cells were plated on 6-well plates, grown in KGM2 medium with 0.05 mM CaCl_2_ until confluent, and then switched to KGM2 with 2 mM CaCl_2_ for 24 h. Thereafter, the cells were treated for 24 h with the recombinant antibodies hAK23 (12.5 µg/mL) or 4B3 (50 µg/mL). As controls, mock incubation (untreated), an isotype-matched hIgG (12.5 µg/mL IgG4, or 50 µg/mL hIgG1), and mAK23 (75 µg/mL) were used. The cells were lysed, and the level of Dsg3, Dsg1 and FcRn was analyzed by Western blot. GAPDH was included as a loading control. A representative experiment is shown. (**b**–**d**) Western blot signals were quantified using ImageJ software, normalized against GAPDH, and expressed as relative values compared to the untreated control. The error bars represent the SD of values obtained from four independent experiments. Statistical analysis was done using one-way analysis of variance (ANOVA) with Dunnett´s post-test. Statistically significant differences are indicated by ** = *p* ≤ 0.01; *** = *p* ≤ 0.001.

Prolonged treatment of keratinocytes with pathogenic anti-Dsg3 antibodies has been shown to result in Dsg3 endocytosis and degradation (see, e.g., [55,56]). Monolayers of hTert keratinocytes were treated with the pathogenic and control antibodies for 24 h, the cells were lysed, and the amount of Dsg3 and Dsg1 was assessed by Western blot. Treatment with the pathogenic anti-Dsg3 antibodies resulted in a reduction of Dsg3 protein amount, as compared to the respective controls (Figure 3a,b). However, the amount of Dsg1 was not reduced (Figure 3a,c), demonstrating that only Dsg3 was degraded after 24 h treatment with the anti-Dsg3 antibodies. The reduction of Dsg3 amount was not due to transcriptional regulation, as demonstrated by the unchanged mRNA levels of Dsg3 (Supplementary Figure S3). The effect on Dsg3 protein amount was both time and dose dependent (Supplementary Figure S4). These data are in accordance with the results of the monolayer dissociation assay (Figure 2).

The effect of the pathogenic anti-Dsg3 antibodies on the level of FcRn was also evaluated by Western blot (Figure 3a). Treatment of hTert keratinocytes with the 4B3 antibody resulted in a highly significant depletion of FcRn, whereas hAK23 and mAK23, as well as the control hIgGs only induced a minor reduction that was not significant (Figure 3d). However, the mRNA amount of FcRn was not significantly altered by any of the antibodies (Supplementary Figure S3).

Taken together, our data show that the reformatted hAK23 and 4B3 antibodies are pathogenic in monolayer dissociation assays, and they induce the characteristic changes in Dsg3 protein level and localization. Furthermore, our findings show that pathogenic anti-Dsg3 antibodies may exhibit a direct effect on FcRn.

### 3.4. Efgartigimod Treatment Prevents the Loss of Monolayer Integrity Induced by hAK23 and 4B3 Monoclonal Antibodies and by PV IgG

Efgartigimod is an engineered human Fc fragment that has been shown to block the binding of IgG type antibodies to FcRn, resulting in reduced IgG antibody half-life [16,40,41,42,43]. To test if blocking of antibody binding to FcRn by efgartigimod has a direct effect on keratinocyte adhesion in vitro, we made use of the cell dissociation assay in the presence of efgartigimod and the pathogenic antibodies hAK23 or 4B3. As controls, we used a wildtype IgG Fc fragment (Fc-WT) that is capable of binding to FcRn, albeit with natural affinity and in a pH-dependent manner. In addition, Fc-IHH, a human IgG Fc fragment carrying three amino acid substitutions (I253A, H310A, H435A) that prevent its binding to FcRn was used as a negative control [32].

**Figure 4:**
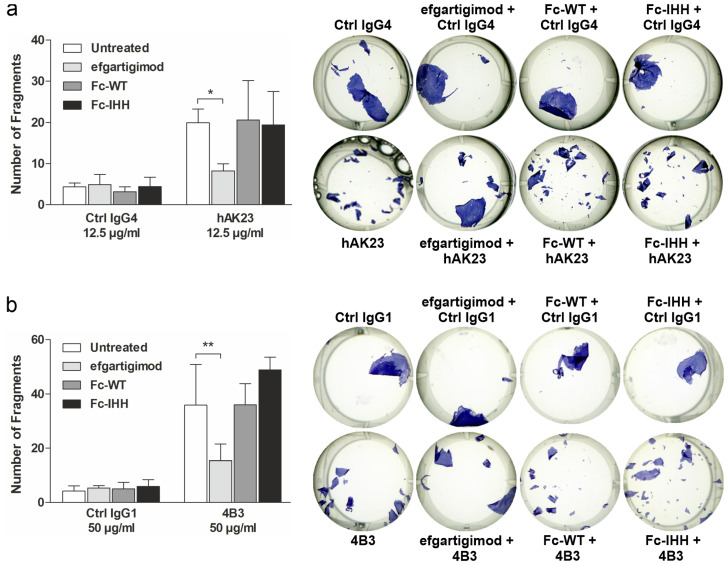
Efgartigimod treatment protects keratinocyte monolayers against dissociation induced by hAK23 and 4B3 anti-Dsg3 antibodies. hTert keratinocytes were plated on 24-well plates, grown in KGM2 medium with 0.05 mM CaCl_2_ until confluent, and then switched to KGM2 with 2 mM CaCl_2_ for 24 h. The cells were left untreated, or efgartigimod, Fc-WT or Fc-IHH (all at final concentration of 25 µg/mL) was applied for 30 min prior to the 24 h treatment with the respective recombinant antibodies: (**a**) hAK23 (12.5 µg/mL) or an isotype-matched hIgG4 control (12.5 µg/mL); or (**b**) 4B3 (50 µg/mL) or an isotype-matched human IgG1 control (50 µg/mL). Monolayer dissociation assay was performed in triplicates, and the number of fragments was quantified using ImageJ software. A representative experiment is shown. The error bars show the SD of the mean values obtained from at least four independent experiments. Statistical analysis was done using two-way ANOVA with Bonferroni’s post-test. Statistically significant differences are indicated by * = *p* ≤ 0.05; ** = *p* ≤ 0.01.

**Figure 5:**
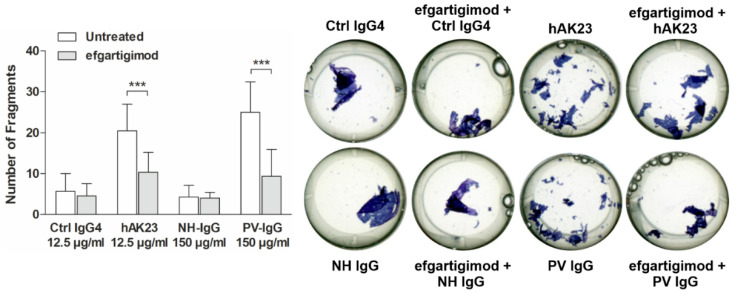
Efgartigimod treatment protects keratinocyte monolayers against dissociation induced by PV IgG. hTert keratinocytes were plated on 24-well plates, grown in KGM2 medium with 0.05 mM CaCl_2_ until confluent, and then switched to KGM2 with 2 mM CaCl_2_ for 24 h. The cells were left untreated, or efgartigimod (25 µg/mL) was applied for 30 min prior to the 24 h treatment with hAK23 (12.5 µg/mL), control IgG4 (12.5 µg/mL), PV IgG (150 µg/mL) or human control IgG (150 µg/mL). Monolayer dissociation assay was performed in triplicates, and the number of fragments was quantified using ImageJ software. A representative experiment is shown. The error bars show the SD of the mean values obtained from five independent experiments. Statistical analysis was done using two-way ANOVA with Bonferroni’s post-test. Statistically significant differences are indicated by *** = *p* ≤ 0.001.

Monolayer fragmentation induced by hAK23 and 4B3 was significantly inhibited by efgartigimod, whereas Fc-WT and Fc-IHH showed no inhibitory effect on monolayer fragmentation (Figure 4a,b). The protective effect of efgartigimod was similar when the cells were pretreated with efgartigimod for 30 min (Figure 4), or when efgartigimod was added 30 min after the pathogenic antibodies (Supplementary Figure S5), and it was dose-dependent (Supplementary Figure S5). Treatment with control hIgG4 combined with either efgartigimod, Fc-WT or Fc-IHH resulted in only a low degree of monolayer fragmentation that was not different from treatment with the control hIgG4 alone (Figure 4a,b). Importantly, efgartigimod was also able to protect the keratinocyte monolayer from fragmentation after treatment with PV IgG derived from a treatment-naïve PV patient with active mucosal-dominant disease (Figure 5). These data show that efgartigimod exhibits a protective effect against anti-Dsg3 antibody- and PV IgG-induced loss of desmosomal adhesion and stabilizes monolayer integrity in human keratinocytes.

### 3.5. Efgartigimod Treatment Does Not Result in Degradation of hAK23 and 4B3 Antibodies

**Figure 6:**
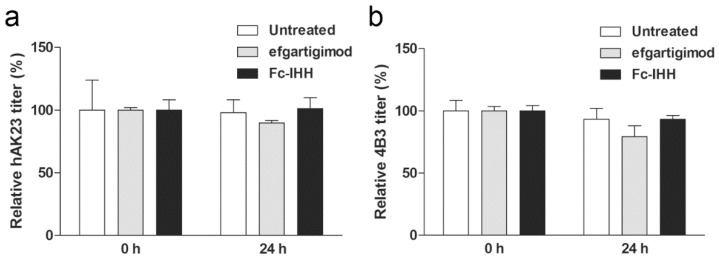
Antibody concentration in the culture medium is not significantly reduced over the assay time, and efgartigimod does not induce significant antibody degradation. Before and after treatment of hTert cells with the pathogenic and control antibodies (see the legend of Figure 4 for details), samples of the media were collected, and the amount of anti-Dsg3 antibodies in the medium was measured with an anti-Dsg3 ELISA. The untreated samples at 0 h were set as 100%, and all other samples were expressed as relative values (%). (**a**) hAK23, and (**b**) 4B3. The error bars show the SD of the mean values obtained from at least five independent experiments. Statistical analysis was done using two-way ANOVA with Bonferroni’s post-test. No significant differences were detected.

Blocking FcRn reduces the recycling of IgG and directs them towards lysosomal degradation. In our assays, this could lead to a reduced availability of the pathogenic IgG and to an apparent reduction of their pathogenic effect. To evaluate this, the amount of hAK23 and 4B3 antibodies in the medium after 24 h incubation in the cell dissociation assay was measured using anti-Dsg3 ELISA. The media of the cells treated with hAK23 or 4B3 together with efgartigimod or Fc-IHH were collected, and the antibody titers in the medium at 0 h (before applying to the cells) and after 24 h were determined by anti-Dsg3 ELISA. The amounts of hAK23 (Figure 6a) or 4B3 (Figure 6b) were not significantly changed in the medium after 24 h. In addition, efgartigimod or Fc-IHH did not induce any significant change in the hAK23 and 4B3 amount in the medium (Figure 6).

These data suggest that FcRn plays a role in the pathogenicity of anti-Dsg3 antibodies in human keratinocytes. Furthermore, FcRn blockade with efgartigimod has a direct protective effect against the loss of desmosomal adhesion in human keratinocytes induced by the pathogenic antibodies in vitro, which is not caused by enhanced degradation of the pathogenic antibodies. Highly efficient transcytosis of efgartigimod across polarized human epithelial T84 cells suggests that efgartigimod can be actively transported across cells and enters tissues. Interestingly, efgartigimod was transported across T84 cells significantly more efficiently than Fc-WT and Fc-IHH fragments, and Fc-WT had a similar transcytosis efficiency as the full-length IgG isotype control or an antibody targeting FcRn via its Fab arms (Supplementary Figure S6). Thus, the protective effect of efgartigimod may also be relevant in vivo in the skin.

### 3.6. Efgartigimod Does Not Rescue Dsg3 Mislocalization Induced by Treatment with hAK23 or 4B3

**Figure 7:**
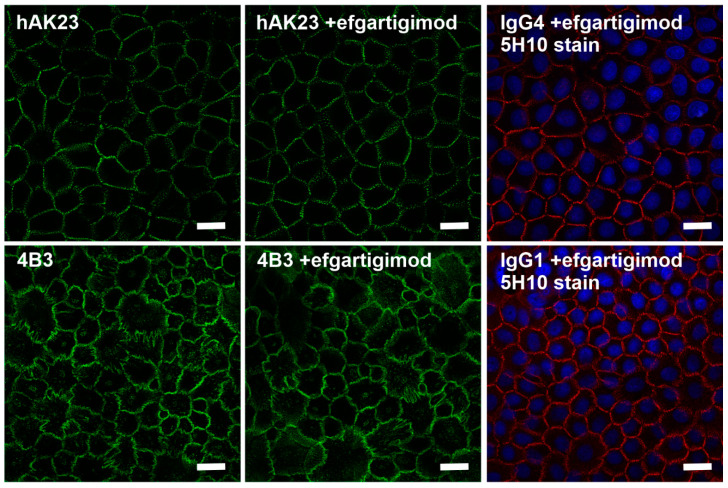
Efgartigimod does not rescue the aberrant Dsg3 staining pattern induced by hAK23 and 4B3. hTert keratinocytes were cultured on coverslips in KGM2 medium with 0.05 mM CaCl_2_ for at least three days, and then switched to KGM2 with 2 mM CaCl_2_ for 24 h. The cells were either mock-treated, or efgartigimod (25 µg/mL) was applied for 30 min, after which a 24 h treatment with the recombinant antibodies hAK23 (12.5 µg/mL), 4B3 (50 µg/mL), an isotype-matched human IgG4 (12.5 µg/mL) or IgG1 control (50 µg/mL) coupled to Alexa Fluor 488 (green) was initiated. After methanol fixation, the control IgG-treated cells were stained with the 5H10 anti-Dsg3 antibody, detected with a fluorochrome coupled secondary antibody (anti-mouse Alexa Fluor 546, red). Representative images from one out of at least three independent experiments are shown. Scale bar: 20 µm.

To further dissect the mechanism of the improvement of monolayer integrity by efgartigimod upon treatment with pathogenic anti-Dsg3 antibodies, hTert cells were treated for 24 h with fluorescently-coupled hAK23, 4B3 or control IgGs (Figure 7). A staining with the 5H10 anti-Dsg3 antibody and A546-coupled secondary antibody (red in Figure 7) was performed with the control IgG-treated samples after fixation to visualize Dsg3. Treatment with efgartigimod did not result in any clear improvement of the disordered Dsg3 staining pattern induced by hAK23 or 4B3 (Figure 7). The control IgG and efgartigimod treated samples show the normal linear staining pattern of Dsg3.

### 3.7. Efgartigimod Treatment Does Not Prevent Dsg3 Degradation Induced by 4B3

**Figure 8:**
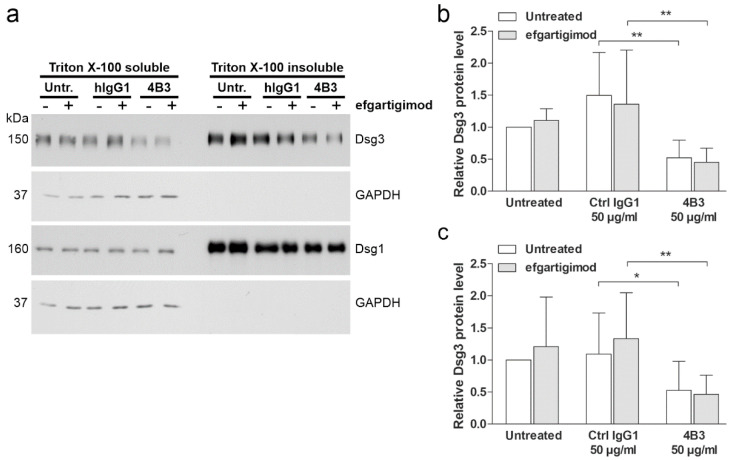
4B3 antibody-induced Dsg3 depletion occurs from the non-desmosomal and desmosomal pools and cannot be rescued by efgartigimod. hTert cells were plated on 6-well plates, grown in KGM2 medium with 0.05 mM CaCl_2_ until confluent, and then switched to KGM2 with 2 mM CaCl_2_ for 24 h. Thereafter, the cells were either left untreated, or efgartigimod (25 µg/mL) was applied for 30 min prior to a 24 h treatment with 4B3 (50 µg/mL) antibodies. As controls, mock incubation (untreated) and an isotype-matched human IgG1 (50 µg/mL) were included. Sequential detergent extraction was performed, resulting in Triton-soluble (non-desmosomal) and Triton-insoluble (desmosomal) pools of proteins. (**a**) Western blot analysis of the fractions was performed to detect Dsg3 and Dsg1. Analysis of GAPDH level was included to ensure equal loading and the purity of the detergent insoluble fraction. Equal percentage of each fraction was loaded. Western blot signals for Dsg3 in the non-desmosomal (**b**) and desmosomal (**c**) pools were quantified using ImageJ software, normalized against GAPDH levels, and expressed as relative amounts compared to the untreated controls. The error bars represent the SD of four independent experiments. Statistical analysis was done using two-way ANOVA with Bonferroni’s post-test. Statistically significant differences are indicated by * = *p* ≤ 0.05, ** = *p* < 0.01.

To study if efgartigimod also displays an effect on the distribution of Dsg3 between the desmosomal and non-desmosomal pools, hTert cells were treated with 4B3 or control hIgG1, and efgartigimod. Desmogleins were fractionated into detergent-soluble (non-desmosomal) and insoluble (desmosomal) pools by extraction with a detergent (Triton X-100) (Figure 8a), as described previously [56,57,58,59].

Treatment with 4B3 resulted in a significant loss of Dsg3 from the non-desmosomal (Figure 8b) and desmosomal (Figure 8c) pools. Furthermore, efgartigimod pretreatment was not able to rescue the loss of Dsg3 from the non-desmosomal and desmosomal pools (Figure 8b,c). In contrast to Dsg3, Dsg1 amount in both pools showed no significant changes with or without efgartigimod treatment (Figure 8a and Supplementary Figure S7). These data suggest that the protective effect of efgartigimod on monolayer integrity is not due to inhibition of antibody-induced Dsg3 degradation.

### 3.8. Efgartigimod Treatment Prevents 4B3-Induced FcRn Degradation

**Figure 9:**
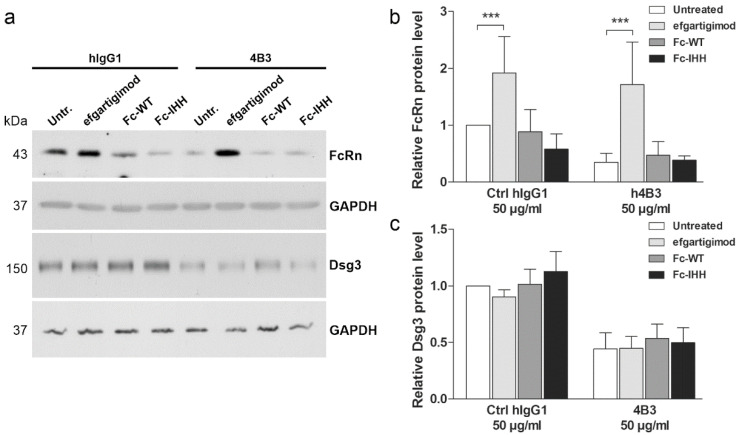
Efgartigimod rescues the 4B3-induced reduction of FcRn but not Dsg3 protein amount. hTert cells were grown on 6-well plates in KGM2 medium with 0.05 mM CaCl_2_ until confluent and then switched to KGM2 with 2 mM CaCl_2_ for 24 h. The cells were either left untreated, or efgartigimod, Fc-WT or Fc-IHH (all at final concentration of 25 µg/mL) were applied for 30 min prior to a 24 h treatment with the 4B3 antibody (50 µg/mL) or an isotype-matched human IgG1 control (50 µg/mL). (**a**) The cells were lysed, and the protein levels of FcRn and Dsg3 were analyzed by Western blot. GAPDH was included as a loading control. Western blot signals of FcRn (**b**) and Dsg3 (**c**) were quantified using ImageJ software, normalized against GAPDH and expressed as relative protein levels compared to the untreated control. The error bars represent the SD of five independent experiments. Statistical analysis was done using two-way ANOVA with Bonferroni’s post-test. Statistically significant differences are indicated by *** = *p* ≤ 0.001.

Since treatment with 4B3, but not with hAK23 or mAK23, resulted in reduction of FcRn protein level (Figure 4), the effects of efgartigimod, Fc-WT and Fc-IHH on FcRn after 4B3 or control hIgG1 treatment were studied (Figure 9). As compared to the hIgG1 treated cells, the protein levels of FcRn and Dsg3 were highly reduced after 4B3 treatment (Figure 9), consistent with the data shown in Figure 4. Efgartigimod treatment resulted in a significant rescue of FcRn level in 4B3-treated cells, whereas Fc-WT and Fc-IHH did not significantly affect FcRn protein level upon 4B3 treatment (Figure 9a,b). However, in hIgG1 treated cells, efgartigimod increased the basal FcRn protein level, whereas treatment with Fc-IHH resulted in a minor but non-significant reduction of FcRn levels. Dsg3 level, on the other hand, was not rescued by efgartigimod, Fc-WT or Fc-IHH (Figure 9c). These data show that efgartigimod may increase the protein level of FcRn in IgG antibody-treated human keratinocytes, whereas the control Fc fragments, Fc-WT and Fc-IHH, are not capable of increasing FcRn protein level.

## 4. Discussion

The main purpose of our study was to explore on the mechanisms of efgartigimod-mediated blockade of FcRn in human keratinocytes treated with anti-Dsg3 antibodies. In a recent phase 2 study of efgartigimod in pemphigus patients, it was shown that efgartigimod exhibits an early onset of action in newly diagnosed and relapsing patients, as evidenced by rapid disease control and potential to reduce the use of corticosteroids [16]. In fact, the PV patient whose serum was used in our study participated in the phase 2 study of efgartigimod [16]. In this PV patient, disease control, defined as no new lesions and established lesions starting to heal, was achieved with a single dose of efgartigimod. Consistently, the patient’s Pemphigus Disease Area Index (PDAI) activity score improved by 91% (baseline PDAI activity 11) by the end of the evaluation period with efgartigimod treatment without concomitant prednisone [16]. These findings are further supported by our data showing that efgartigimod treatment was able to prevent the monolayer fragmentation of keratinocytes treated with PV IgG fraction of the said patient.

So far, the therapeutic effect of efgartigimod in IgG-mediated autoimmune diseases has been thought to rely on the blockade of IgG autoantibody binding to FcRn in endosomes, resulting in degradation of the pathogenic antibodies and reduction of their serum concentrations [40,41,42,43]. However, it has also been shown that FcRn is expressed in human epidermal keratinocytes and in numerous epithelial cell types [32,33,39,60,61,62], in which FcRn has been suggested to be involved in the endocytic trafficking and transcytosis of IgG, even though FcRn may mediate different trafficking steps in a cell type-specific manner (see, e.g., [60,63,64]). In pemphigus, FcRn-mediated uptake and endocytic trafficking of autoantibodies targeted towards non-desmoglein antigens, including mitochondrial proteins, has been postulated to result in mitochondrial damage and apoptosis, highlighting FcRn as a potential therapeutic target in pemphigus [60].

Monoclonal anti-Dsg3 antibodies, especially mAK23, are frequently used for studies on the molecular pathogenesis of pemphigus in cell culture, but mouse antibodies are not capable of interacting with human FcRn, and are therefore not suitable for studying the role of FcRn in pemphigus pathogenesis in human cells [12]. To elucidate the function of FcRn and its blocking agent efgartigimod in human keratinocytes, we have generated novel recombinant anti-Dsg3 antibody tools that contain a human Fc region capable of interacting with FcRn in combination with pathogenic anti-Dsg3 reactivity. Our chimeric hAK23 antibody contains a human IgG4 Fc region and a mouse Fab region that is identical with the established mouse anti-Dsg3 mAK23 antibody. Importantly, hAK23 antibody reduced the keratinocyte monolayer integrity, demonstrating its pathogenicity. Furthermore, changes in the organization of Dsg3 at the plasma membrane, together with a profound loss of Dsg3 protein level upon 24 h treatment of hTert keratinocytes with hAK23 were observed. In comparison to the parental mAK23, lesser amounts of hAK23 were needed to induce the same pathogenic effects, suggesting that interaction of hAK23 with human FcRn, which is not possible for mAK23, may potentiate the pathogenicity of the autoantibodies. The IgG subtype does not appear to considerably affect the pathogenicity, as we have obtained similar data with IgG4 (this manuscript) and IgG1 (data not shown) versions of hAK23. In addition, the recombinant, fully human anti-Dsg3 IgG1 antibody 4B3, originally derived from a PV patient, showed a similar pathogenic profile. Thus, our novel, reformatted hAK23 and 4B3 antibodies represent relevant and valuable monoclonal antibody tools to study the pathomechanisms of pemphigus in human keratinocytes.

Our data show that the effect of pathogenic anti-Dsg3 antibodies and PV IgG on keratinocyte adhesion was ameliorated by blocking FcRn-IgG interactions with efgartigimod, as the monolayer integrity in the dissociation assay was restored in the presence of efgartigimod, despite treatment with the pathogenic antibodies or PV IgG. However, efgartigimod was not capable of rescuing the loss of Dsg3 or the Dsg3 rearrangement at the plasma membrane. Therefore, the improvement of keratinocyte adhesion by efgartigimod upon anti-Dsg3 treatment seems not to be based on a direct effect on Dsg3 localization or amount. In addition, efgartigimod did not substantially increase the degradation of the pathogenic antibodies in our assays, as the antibody titers in the medium remained constant over the assay time. Therefore, our data suggest a possible novel, beneficial effect of FcRn blockade by efgartigimod on keratinocyte adhesion that is different from the role of FcRn in IgG recycling.

Surprisingly, treatment of keratinocytes with efgartigimod in combination with non-pathogenic IgG antibodies resulted in an increase in the protein level of FcRn, without change of FcRn mRNA level. This was observed both with the pathogenic anti-Dsg3 antibodies and control IgG, whereas Fc-WT and Fc-IHH did not enhance FcRn protein level. Therefore, there appears to be a positive regulatory effect of efgartigimod on FcRn protein level in human keratinocytes treated with IgG antibodies.

Treatment with 4B3, but not with hAK23, mAK23 or the control IgGs, resulted in a significant reduction of FcRn level in human keratinocytes. However, efgartigimod was able to rescue the FcRn protein level upon 4B3 treatment. It is not clear why a loss of FcRn is induced only by the 4B3 antibody, but not by the other anti-Dsg3 antibodies, although the Dsg3 protein level was reduced by all anti-Dsg3 antibodies. We do not think that this is due to the antibody concentrations used (50 µg/mL for 4B3 and 12.5 µg/mL for hAK23), since they were chosen based on the pathogenic effect of the respective antibodies in the monolayer fragmentation assay.

One possible explanation for the different effects on FcRn protein level is that although the epitopes of both hAK23 and 4B3 reside in the EC1 ectodomain of Dsg3, they are not completely overlapping and may thus induce different downstream effects [10,11]. In addition, 4B3 is not completely Dsg3 specific in human cells, as it has been shown to be cross-reactive with human Dsg1 [11]. Therefore, it is possible that the capability of 4B3 to crosslink Dsg3 and Dsg1 results in different kind of downstream effects, as compared to fully Dsg3-specific antibodies. FcRn is involved in the degradative transport of antibody–antigen complexes, and larger FcRn-IC complexes that contain Dsg3 and Dsg1 may more efficiently be directed to degradation in lysosomes than those that only contain Dsg3 [37,65]. This would be consistent with the findings showing that ICs with multiple IgG molecules bound to their target antigens direct FcRn to lysosomes, in contrast to monovalent IgG that are recycled back to the cell surface [37,65].

Another explanation for the 4B3 specific effects could be a different Dsg-mediated downstream signaling response. It has been shown that antisera from pemphigus patients display different effects on signaling cascades, depending on the presence of Dsg1 antibodies [53], and these signaling responses could affect FcRn trafficking and degradation. PV IgG often contain a mixture of antibodies with varying antigen specificities, which may include antibodies against desmocollins, mitochondrial proteins, acetylcholine receptors and other antigens [3,60]. Therefore, monoclonal antibodies may not be capable of inducing all of the signaling responses that are observed upon PV IgG treatment. Furthermore, loss of Dsg3 after anti-Dsg3 treatment may even not be the key event per se that leads to the cellular responses that include diverse signaling cascades and even activation of apoptotic pathways (reviewed in [8]).

The mechanism of the increase or rescue of FcRn protein level by efgartigimod in 4B3-treated cells is not clear, but signaling pathways and cytokines can down-regulate FcRn expression [63,66]. In Hashimoto thyreoiditis, another IgG-mediated autoimmune disorder, FcRn expression in thyroid epithelial cells was shown to be reduced, and interferon-γ (IFN-γ), tumor necrosis factor-α, and interleukin-4 or -10 treatment diminished FcRn expression [63]. IFN-γ treatment results in the activation of the Janus tyrosine kinases, JAK1 and JAK2, that recruit and activate the transcription factor STAT1 (signal transducer and activator of transcription 1), which was shown to repress FcRn expression [66]. Cytokines, such as IFN-γ, play a role in autoimmune diseases, including pemphigus, and the levels of some cytokines are elevated in the sera and blister fluid of pemphigus patients [67]. IFN-γ has also been shown to impair the transcytosis of IgG in polarized lung epithelial cells [66], indicating that regulation of FcRn by cytokines in autoimmune diseases may be relevant also for the pathogenesis of pemphigus.

In the light of our data, we propose that the function of FcRn in keratinocytes may be more diverse than its known effect in endothelial cells (i.e., antibody recycling). It has been shown that in some tissues, such as the intestine, FcRn is important for the transcytosis of IgG through the epithelia, whereas FcRn-mediated transcytosis through endothelial cells may be a prerequisite for autoantibodies to gain access to their target tissues (reviewed in [36,38]). Therefore, we propose that in tissues such as the epidermis, FcRn may function in “seeding immunity” by transporting the soluble, monomeric autoantibodies towards the autoantibody target cells, epidermal keratinocytes in pemphigus. This hypothesis is supported by previous studies. Biodistribution studies in mice have shown a decrease in the tissue-to-blood ratio of the autoantibodies in the epidermis in FcRn knockout mice compared to wild type mice [68,69]. This directly suggests that FcRn may play a role in transporting IgG from blood circulation to skin. Further studies also suggest that FcRn is critical for target site access by autoantibodies. Li et al. showed that in murine models injected with antibodies against BP180, Dsg1, and Dsg3, FcRn deficient mice were resistant to bullous pemphigoid, pemphigus foliaceus and PV, respectively [70]. These studies, together with our data, indicate that in addition to an effect on autoantibody degradation, target site access of autoantibodies may be a further aspect mediated by FcRn in these diseases. According to this hypothesis, the distribution of anti-Dsg autoantibodies in the epidermis by FcRn would be prevented by efgartigimod, resulting in reduced pathogenicity of the autoantibodies in the target tissues and in an improvement of desmosomal cell adhesion. Importantly, efgartigimod exerts a direct protective effect on keratinocyte adhesion even in the presence of pathogenic anti-Dsg antibodies, even though the exact mechanisms of stabilization of keratinocyte adhesion still need to be elucidated in detail.

## 5. Conclusions

This study is the first demonstration of a direct effect of FcRn blockade by efgartigimod on the main anti-Dsg autoantibody target cells in pemphigus, the epidermal keratinocytes. This direct effect may have very important implications for the treatment of pemphigus, as it extends the potential mode of action of efgartigimod from the blocking of antibody recycling in the blood to more immediate and direct effects on keratinocyte adhesion. This effect is likely to translate in vivo, as supported by the efficient transcytosis of efgartigimod observed in our in vitro assay. Such effects may also explain why a rapid improvement of skin lesions is observed in pemphigus patients treated with efgartigimod in the presence of circulating anti-desmoglein antibodies.

In the future, patient sera with IgG antibodies or reformatted monoclonal antibodies with a human Fc region will facilitate detailed investigations on the role of FcRn in the pathogenic action of autoantibodies in pemphigus, but also in further IgG-mediated autoimmune diseases. Such studies will increase our understanding of the pathogenic mechanisms of autoantibodies and shed light on the potential of IgG-targeted therapies. Importantly, our data show that keratinocyte adhesion can be stabilized by FcRn targeting entities even in the presence of pathogenic antibodies, which may provide a novel treatment paradigm for pemphigus.

## Data Availability

The data are available from the corresponding author upon a reasonable request.

## Supplementary Materials

The following are available online at https://www.mdpi.com/2073-4409/11/6/942/s1, Figure S1: Apparent affinities and potencies in competition ELISA for anti-Dsg3 antibodies used in this study; Figure S2: Pathogenic effect of hAK23 and 4B3 on Dsg3 localization; Figure S3: Treatment of keratinocytes with the recombinant anti-Dsg3 antibodies does not affect mRNA levels of Dsg3 and FcRn; Figure S4: Treatment of keratinocytes with the recombinant hAK23 antibody results in Dsg3 depletion in a time- and dose-dependent manner; Figure S5: Efgartigimod treatment protects the monolayers against dissociation induced by 4B3 anti-Dsg3 antibodies; Figure S6: Efgartigimod is efficiently transcytosed from the apical to the basolateral side of intestinal epithelial T84 cells; Figure S7: Anti-Dsg3 4B3 antibody or efgartigimod treatment does not result in changes in Dsg1 protein level.
