# Correction: Zakrzewicz et al. Stabilization of Keratinocyte Monolayer Integrity in the Presence of Anti-Desmoglein-3 Antibodies through FcRn Blockade with Efgartigimod: Novel Treatment Paradigm for Pemphigus? *Cells* 2022, *11*, 942

**DOI:** 10.3390/cells11101700

**Published:** 2022-05-20

**Authors:** Anna Zakrzewicz, Celina Würth, Benedikt Beckert, Simon Feldhoff, Katrien Vanderheyden, Stian Foss, Jan Terje Andersen, Hans de Haard, Peter Verheesen, Vladimir Bobkov, Ritva Tikkanen

**Affiliations:** 1 Institute of Biochemistry, Medical Faculty, University of Giessen, Friedrichstrasse 24, 35392 Giessen, Germany; anna.zakrzewicz@chiru.med.uni-giessen.de (A.Z.); celina.wuerth@dentist.med.uni-giessen.de (C.W.); benedikt.beckert@biochemie.med.uni-giessen.de (B.B.); simon.feldhoff@bio.uni-giessen.de (S.F.); 2 Argenx BV, Industriepark Zwijnaarde 7, 9052 Ghent, Belgium; kvanderheyden@argenx.com (K.V.); hdehaard@argenx.com (H.d.H.); pverheesen@argenx.com (P.V.); vbobkov@argenx.com (V.B.); 3 Department of Immunology, University of Oslo and Oslo University Hospital, Rikshospitalet, 0372 Oslo, Norway; stian.foss@medisin.uio.no (S.F.); j.t.andersen@medisin.uio.no (J.T.A.); 4 Institute of Clinical Medicine and Department of Pharmacology, University of Oslo and Oslo University Hospital, 0372 Oslo, Norway

The authors wish to make the following changes to their paper [1]. Figure 4 should be replaced. In the original publication, there was a mistake in Figure 4a as published. One of the images showing the monolayer dissociation assay examples was accidentally duplicated (Fc-WT + hAK23 and Fc-IHH + hAK23 were duplicated). We have now replaced Fc-WT + hAK23 with the correct image. The corrected Figure 4 appears below. 


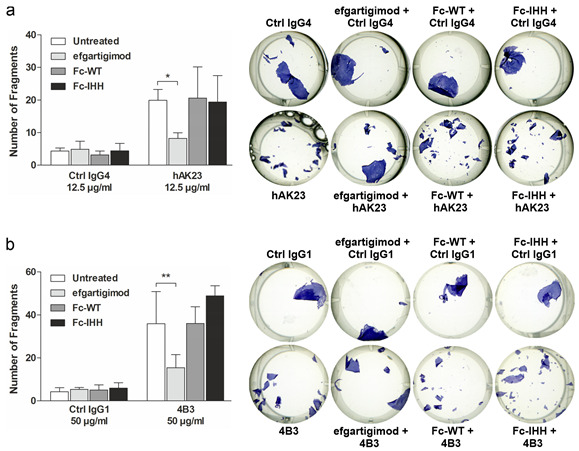


The authors apologize for any inconvenience caused and state that the scientific conclusions are unaffected. This correction was approved by the Academic Editor. The original publication has also been updated.
