# Supplementary material for: Stabilization of Keratinocyte Monolayer Integrity in the Presence of Anti-Desmoglein-3 Antibodies through FcRn Blockade with Efgartigimod: Novel Treatment Paradigm for Pemphigus?
Source: Cells. 2022 Mar 10;11(6):942. doi: 10.3390/cells11060942 (PMC8946243; doi:10.3390/cells11060942)
Supplement: Supplementary file 1 [file cells-11-00942-s001.zip › cells-1504809-supplementary.pdf]

Supplementary Figure S1

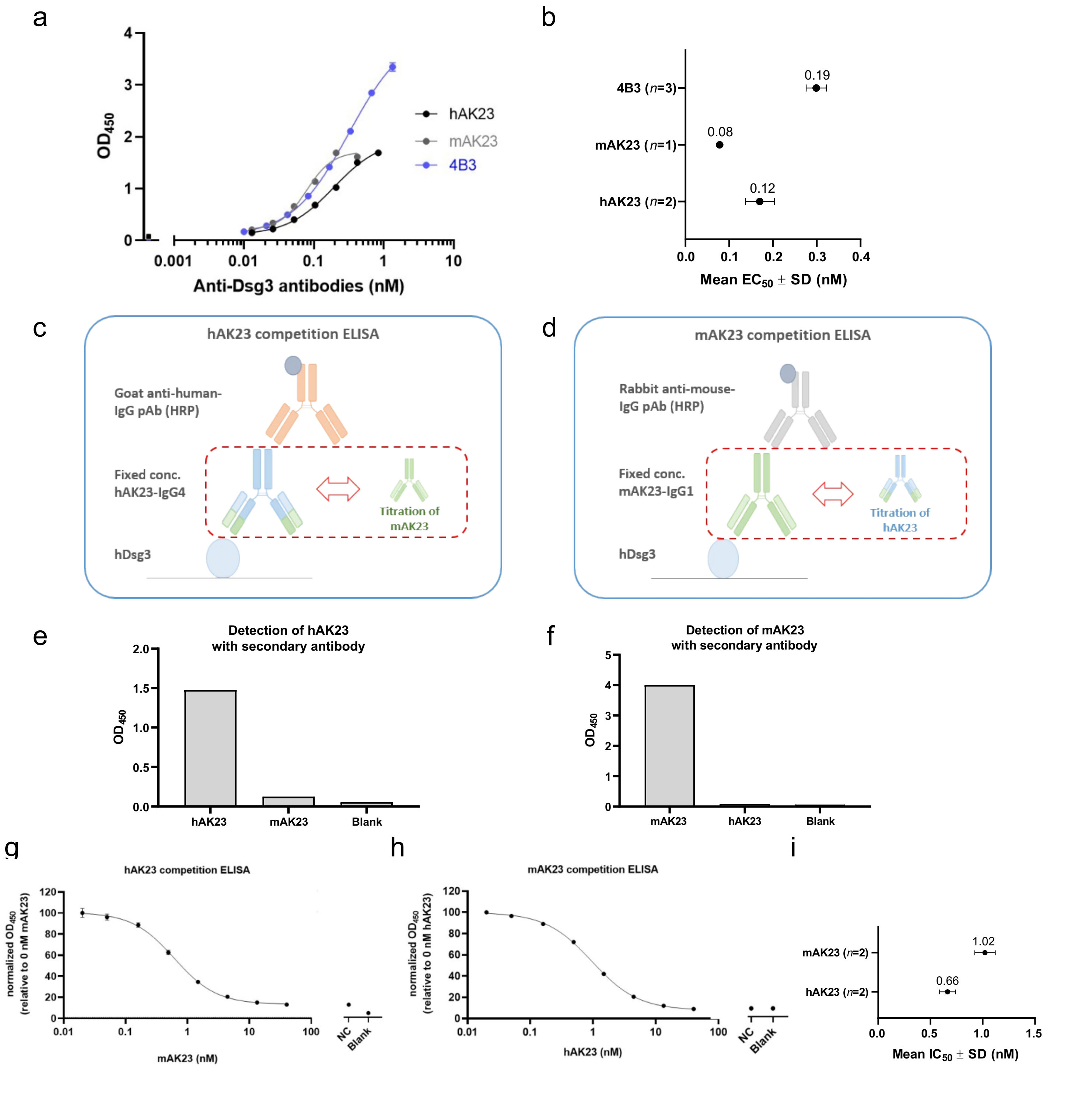

Supplementary Figure S1. Apparent affini es and potencies in compe on ELISA for an -Dsg3 an bodies used in this study. 4B3, hAK23, and mAK23 an bodies show similar apparent affinity (EC_{50}) to human Dsg3 in the MESACUP-2 Dsg3 ELISA assay. (**a**) Dose-response curves show the binding of 4B3, hAK23, and mAK23 antibodies to human Dsg3. Data points show the mean ± SD of two technical replicates from one representative experiment for a titration series of hAK23 (0.013 nM to 0.833 nM), mAK23 (0.013 nM to 0.417 nM), and 4B3 (0.010 nM to 1.333 nM).(**b**) Mean EC_{50} values ± SD of 1-3 independent experiments. (**c**) Schematic of the competition ELISA for hAK23. (**d**) Schematic of the competition ELISA for mAK23. **(e-(f**) Secondary antibodies to detect hAK23 and mAK23 competition in ELISA did not show cross-reactivity to coating and competing antibody. **g-i**) Reformatted hAK23 and its parental version mAK23 compete for binding to human Dsg3 with similar affinities. (**g**) Dose-response curves show inhibition of hAK23 binding at a fixed concentration (tracer) to human Dsg3 by increased concentrations of its parental version mAK23 (competing antibody), (**h**) inhibition of mAK23 (tracer) binding by its reformatted version hAK23 (competing antibody). Data points show the normalized mean ± SD of two technical replicates from one representative experiment. Data are normalized to maximal binding of a tracer antibody (100%) without competing antibody. The negative control (NC) and blank show binding signals of saturating concentrations of competing antibody and assay diluent, respectively, without a tracer antibody. **(i)** Mean IC_{50} values ± SD for hAK23 and mAK23 from two independent competition ELISA experiments, each performed at least in duplicate.

Supplementary Figure S2

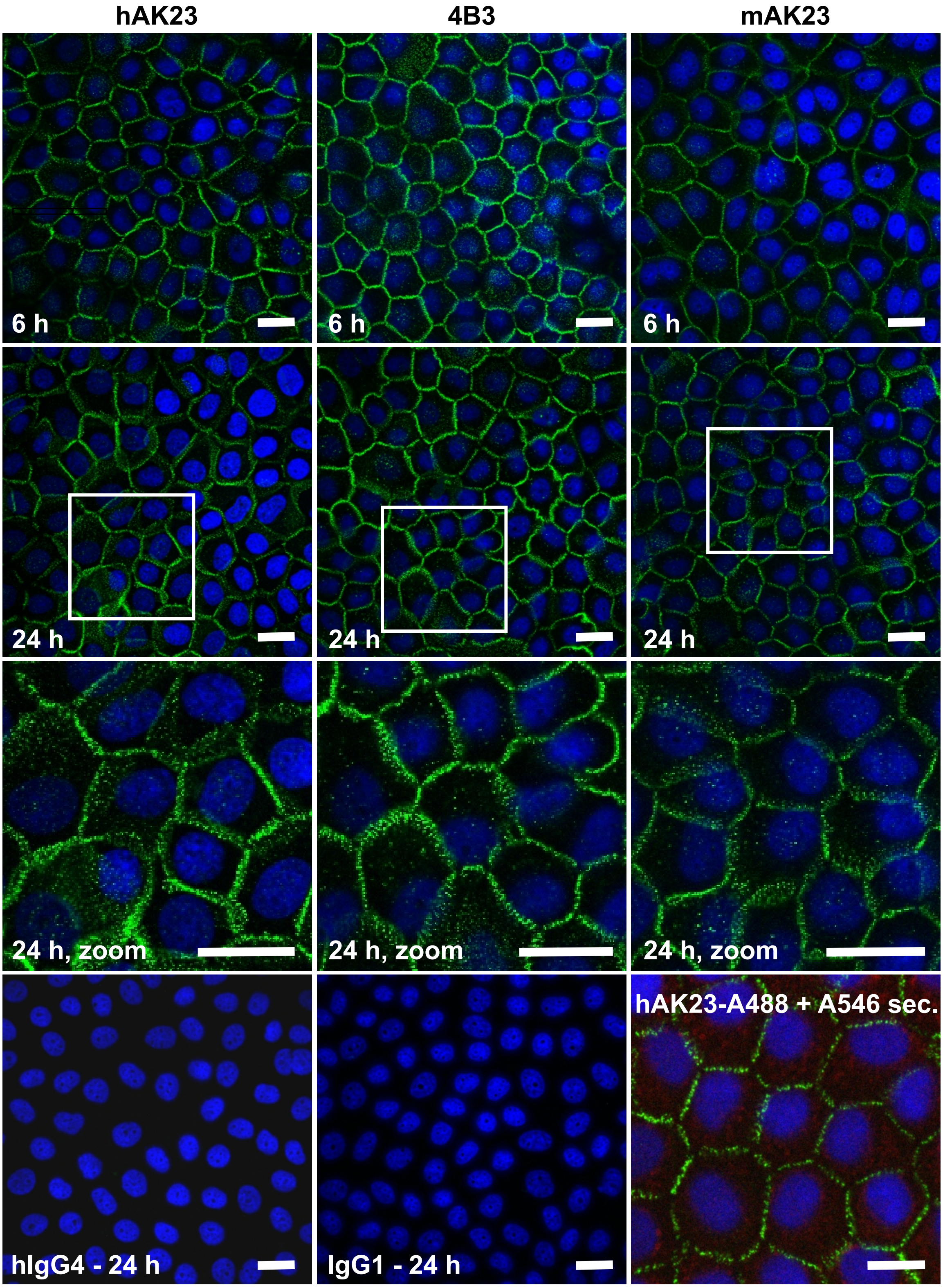

Supplementary Figure S2. Pathogenic effect of hAK23 and 4B3 on Dsg3 localiza on. hTert kera nocytes were cultured on coverslips in KGM2 medium with 0.05 mM $CaCl_2$ for at least three days, and then switched to KGM2 with 2 mM $CaCl_2$ for 24 h. Thereafter, the cells were treated with recombinant an -Dsg3 an bodies (hAK23 or 4B3) for further 6 h or 24 h. Treatment with mAK23 or an isotype-matched hIgG (hIgG1 and hIgG4) was included as control. After methanol fixation, fluorochrome-coupled secondary antibodies (anti-mouse or anti-human Alexa Fluor 488) were used for the detection of the Dsg3-bound primary antibodies. The coverslips were mounted using a mounting medium with DAPI (blue). The third row shows a magnification of the white squares on row 2. The last image shows the secondary antibody (red) control for Figure 1b. Representative images selected from one of at least three independent experiments are shown. Scale bar: 20 µm.

Supplementary Figure S3

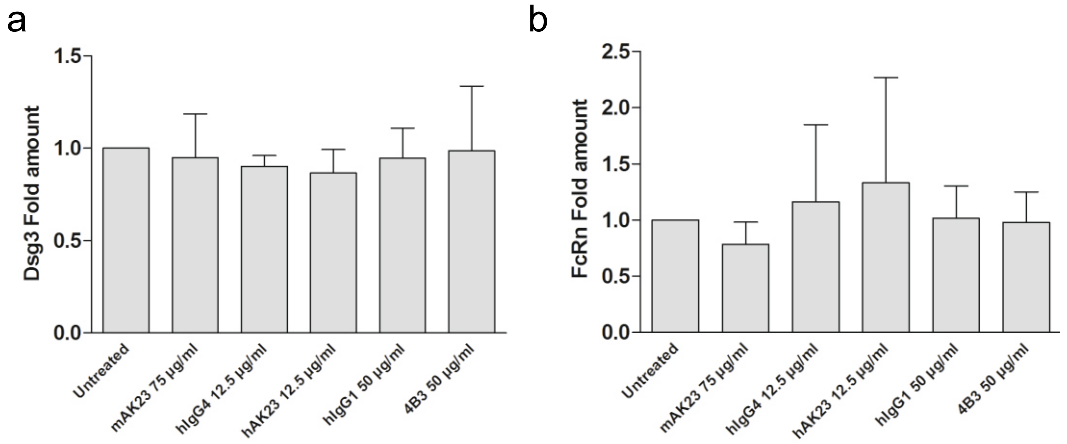

Supplementary Figure S3. Treatment of keratinocytes with the recombinant anti-Dsg3 antibodies does not affect mRNA levels of Dsg3 and FcRn. hTert cells were plated on 6 well plates, grown in KGM2 medium with 0.05 mM $CaCl_2$ until confluent and then switched to KGM2 with 2 mM $CaCl_2$ for 24 h. Thereafter, the cells were treated for 24 h with the recombinant antibodies hAK23 (12.5 µg/ml) or 4B3 (50 µg/ml). As controls, mock incubation (untreated), an isotype-matched hIgG (IgG4 12.5 µg/ml or IgG1 50 µg/ml) and mAK23 (75 µg/ml) were used. RNA was isolated from the cells, and the mRNA level of Dsg3 (**a**) and FcRn (**b**) was assessed by quantitative real-time PCR. The error bars show the SD of three independent experiments. Statistical analysis was done using One-way ANOVA with Dunnett´s post-test. No statistical significances were observed.

Supplementary Figure S4

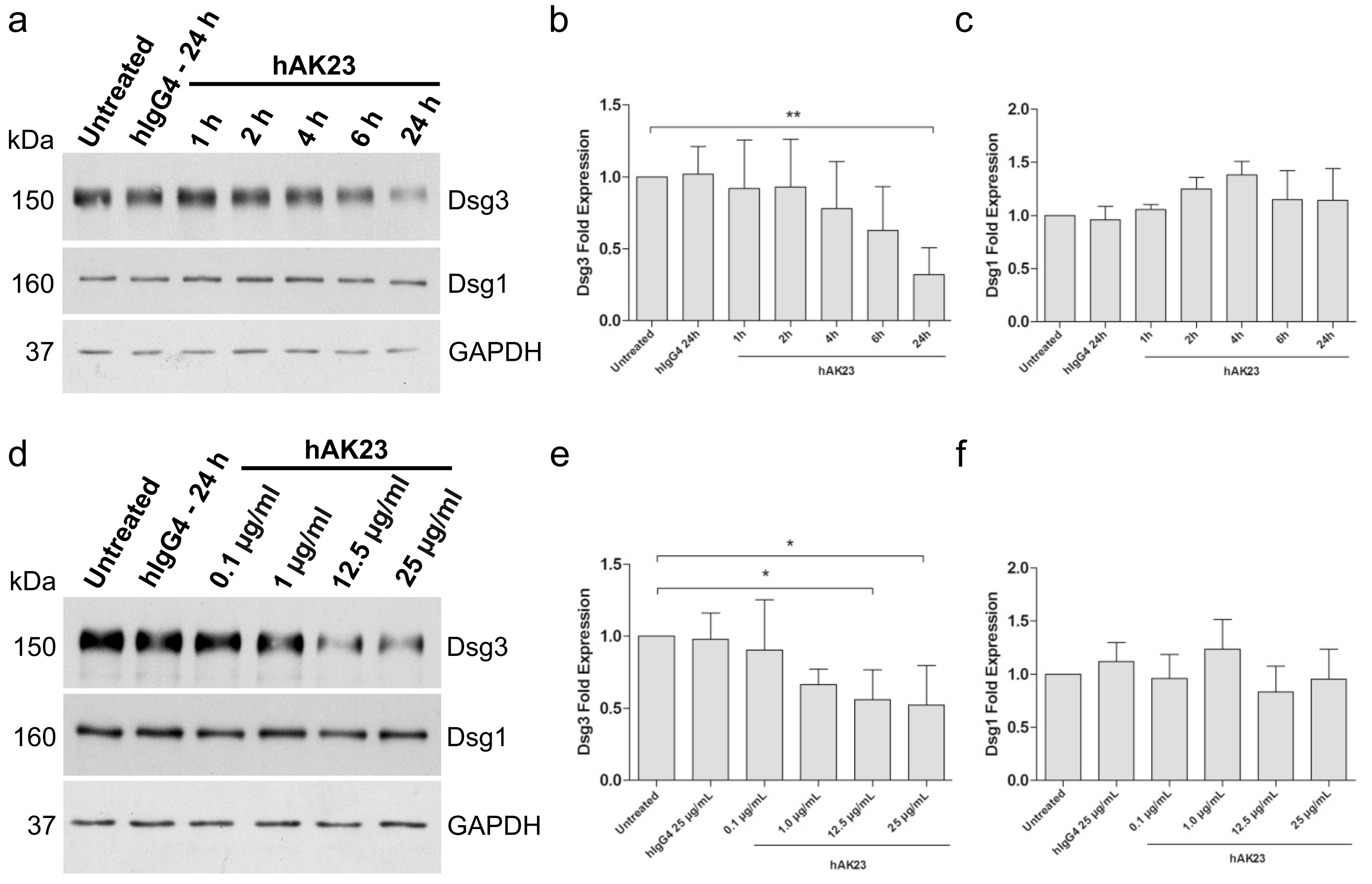

Supplementary Figure S4. Treatment of keratinocytes with the recombinant hAK23 antibody results in Dsg3 depletion in a time- and dose-dependent manner. (**a**) hTert cells were grown in KGM2 medium with 0.05 mM $CaCl_2$ until confluent and then switched to KGM2 with 2 mM $CaCl_2$ for 24 h. Thereafter, the cells were treated for the indicated times with hAK23 (12.5 µg/ml). As controls, mock incubation (untreated) and an isotype-matched IgG4 (12.5 µg) treatment for 24 h were used. The cells were lysed and the level of Dsg3 and Dsg1 was analyzed by Western blot. GAPDH was used as a loading control. Representative Western blots of one experiment are shown. (**b,c**) The Western blot signals were quantified using ImageJ software, normalized against GAPDH and expressed as relative values to the untreated control. (**d**) Confluent hTert cells after 24 h culture in high calcium medium were treated with different amounts of hAK23 (0.1 up to 25 µg/ml) antibodies for further 24 h. As controls, mock incubation (untreated) and an isotype-matched IgG4 (25 µg) were used. The cells were lysed and the level of Dsg3 and Dsg1 was analyzed by Western blot. GAPDH was used as a loading control. Representative images of Western blots from one experiment are shown. (**e,f**) Western blot signals were quantified using ImageJ software, normalized against GAPDH and expressed as relative values to the untreated control. The error bars represent SD of values obtained from at least three independent experiments. Statistical analysis was done using One-way analysis of variance (ANOVA) with Dunnett´s post-test. Statistically significant differences are indicated by * = $p \leq 0.05$; ** = $p \leq 0.01$. .

Supplementary Figure S5

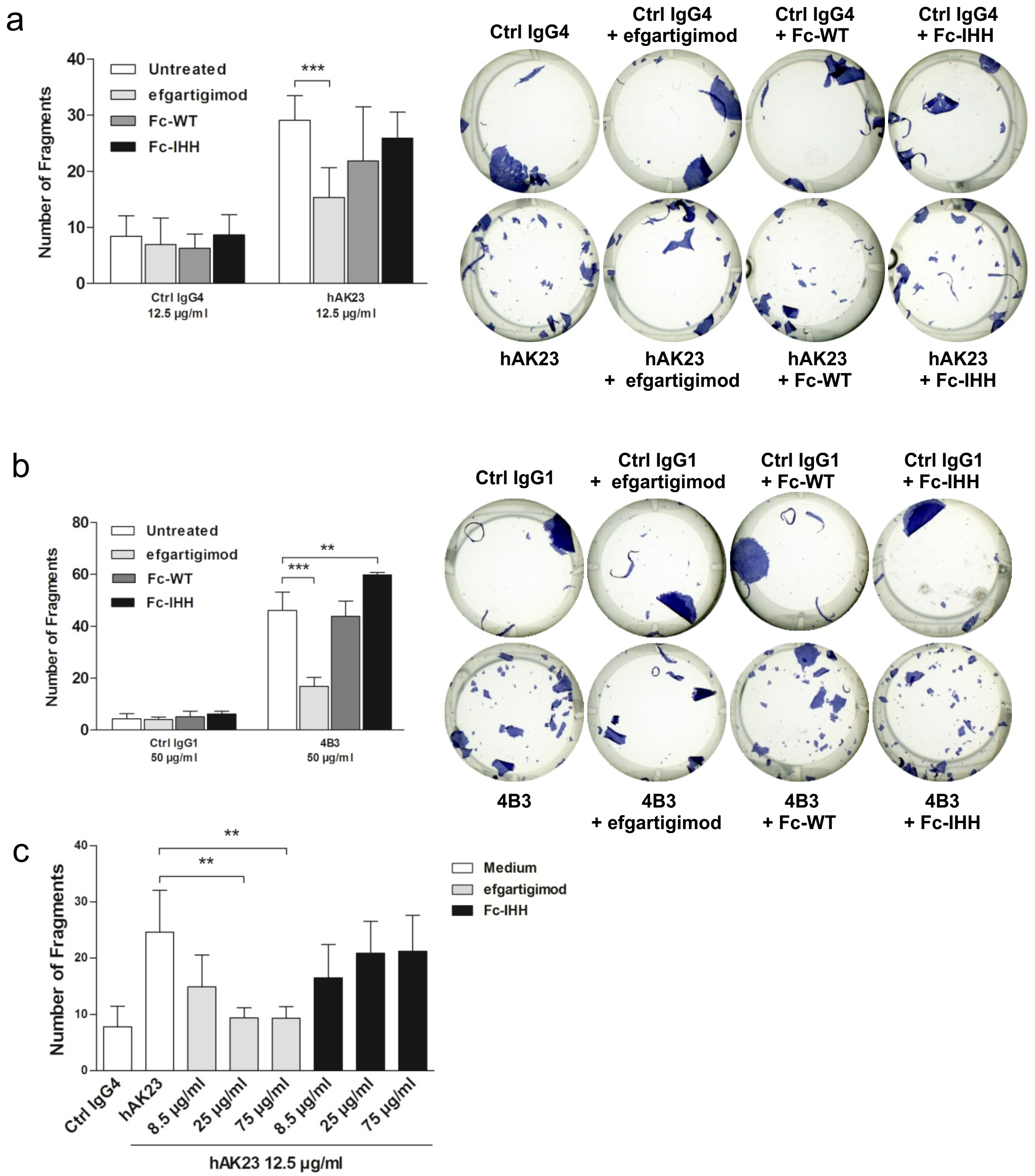

Supplementary Figure S5. Efgartigimod treatment protects the monolayers against dissociation induced by anti-Dsg3 antibodies. hTert cells were plated in 24 well plates, grown in KGM2 medium with 0.05 mM $CaCl_2$ until confluent and then switched to KGM2 with 2 mM $CaCl_2$ for 24 h. The cells were either left untreated, or efgartigimod, Fc-WT or Fc-IHH (all at final concentration of 25 µg/mL) was applied 30 min after addition of the recombinant antibodies, followed by a 24 h incubation. (**a**) hAK23 (12.5 µg/ml) or an isotype-matched hIgG4 control (12.5 µg/ml). (**b**) 4B3 (50 µg/ml) or an isotype-matched human IgG1 control (50 µg/ml). (**c**) Dose-dependent effect of efgartigimod upon hAK23 treatment. Monolayer integrity assay was performed in triplicates, and the number of fragments was quantified using the ImageJ software. Representative images of one experiment are shown. The error bar represents the SD of the mean values obtained from at least three independent experiments. Statistical analysis was done using Two-way ANOVA with Bonferroni's post-test. Statistically significant differences are indicated by ** = $p \leq 0.01$; *** = $p \leq 0.001$.

Supplementary Figure S6

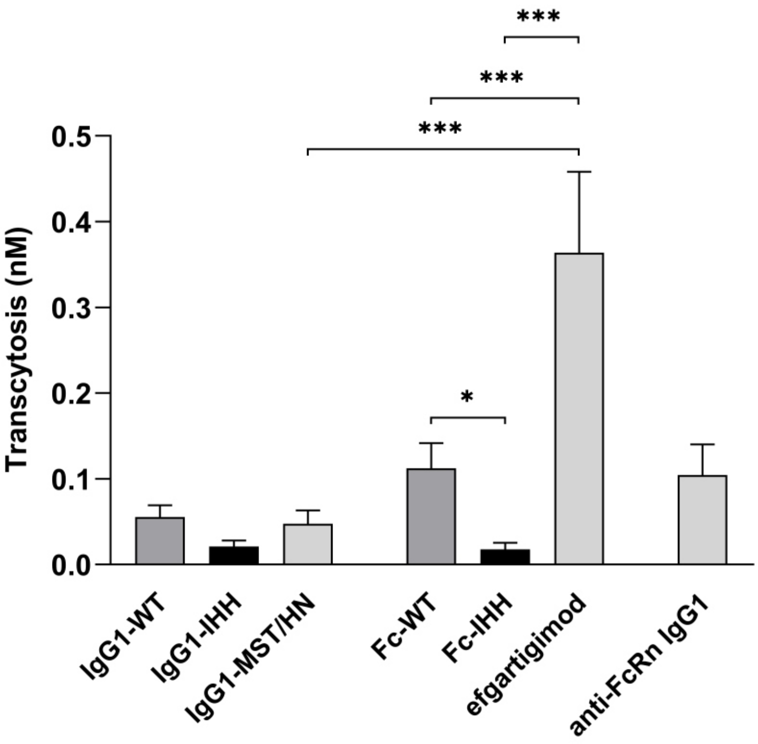

Supplementary Figure S6. Efgartigimod is efficiently transcytosed from the apical to the basolateral side of intestinal epithelial T84 cells. Human epithelial T84 cells were grown on collagen-coated Transwell filters until confluent and monitored by measuring the trans-epithelial resistance. After 5 to 7 days, 200 nM IgG1-WT, IgG1-IHH, IgG1-MST-HN, Fc-WT, Fc-IHH, efgartigimod, or an Anti-FcRn IgG1 were added in HBSS on the apical side and incubated for 4 h. The basolateral media were collected, and the transcytosed full-length IgGs or Fc fragments were measured by enzyme-linked immunosorbent assay (ELISA) and plotted against standard curves for each antibody or Fc fragment. The error bars show the SD of at least three independent experiments for IgG1-WT, IgG1-IHH, IgG1-MST-HN, Fc-WT, Fc-IHH and efgartigimod. Statistical analysis was done using Two-way ANOVA with Bonferroni's post-test. Statistically significant differences are indicated by * = $p \leq 0.05$; *** = $p \leq 0.001$. Two independent experiments were carried out with the anti-FcRn IgG1, for which no statistical analysis was performed. The anti-FcRn IgG1 was previously described in Nixon et al., Front Immunol. 2015; 6:176, and the sequences for the heavy and light chains were retrieved from the patent application WO2012/167039.

Supplementary Figure S7

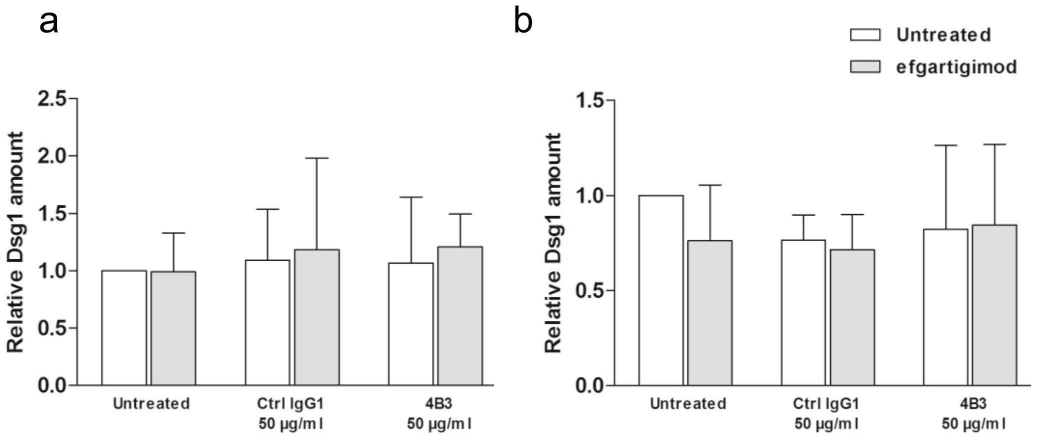

Supplementary Figure S7. Anti-Dsg3 4B3 antibody or efgartigimod treatment does not result in changes in Dsg1 protein level. hTert cells were plated on 6 well plates, grown in KGM2 medium with 0.05 mM $CaCl_2$ until confluent and then switched to KGM2 with 2 mM $CaCl_2$ for 24 h. Thereafter, the cells were either left untreated, or efgartigimod (25 mg/ml) was applied for 30 min prior to a 24 h treatment with 4B3 (50 µg/ml) antibodies. As controls, mock incubation (untreated) and an isotype-matched human IgG1 (50 µg/ml) were included. Sequential detergent extraction was performed, resulting in Triton X -100 soluble (non-desmosomal) and Triton X-insoluble (desmosomal) pools of proteins. Quantification of the Dsg1 Western blots of the (**a**) non-desmosomal fractions and (**b**) desmosomal fractions. The signals were quantified using ImageJ software, normalized against GAPDH levels, and expressed as relative amounts compared to the untreated controls. The error bars represent the SD of the values obtained from four independent experiments. Statistical analysis was done using Two-way ANOVA with Bonferroni's post-test. No statistical significance was observed.
